# Direct Anchoring of Molybdenum Sulfide Molecular Catalysts on Antimony Selenide Photocathodes for Solar Hydrogen Production

**DOI:** 10.1021/acsenergylett.4c01570

**Published:** 2024-07-12

**Authors:** Pardis Adams, Jan Bühler, Iva Walz, Thomas Moehl, Helena Roithmeyer, Olivier Blacque, Nicolò Comini, J. Trey Diulus, Roger Alberto, Sebastian Siol, Mirjana Dimitrievska, Zbynek Novotny, S. David Tilley

**Affiliations:** † Department of Chemistry, University of Zurich, Zurich, 8057, Switzerland; ‡ Department of Physics, University of Zurich, Zurich, 8057, Switzerland; § Swiss Light Source, Paul Scherrer Institute, Villigen, 5232, Switzerland; ∥ Surface Science and Coating Technologies Laboratory/Transport at Nanoscale Interfaces Laboratory, Swiss Federal Laboratories for Materials Science and Technology (EMPA), Dübendorf, 8600, Switzerland

## Abstract

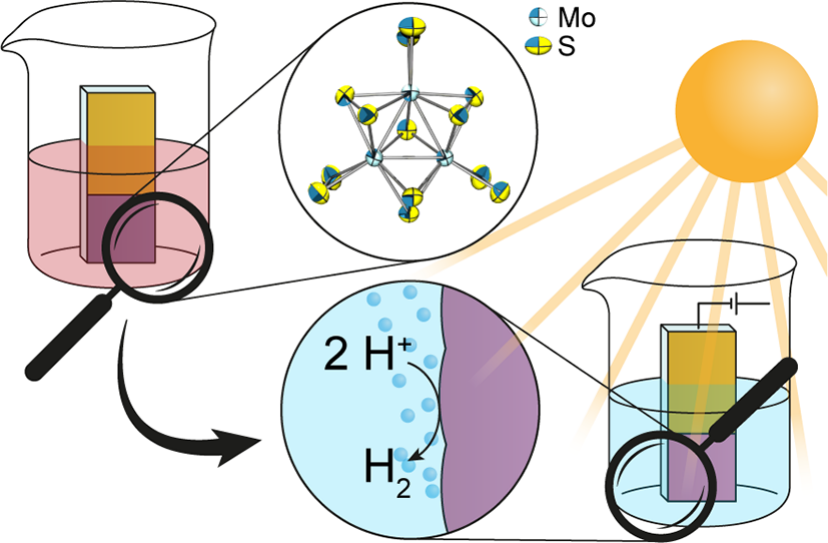

Molybdenum sulfide serves as an effective nonprecious metal catalyst for hydrogen evolution, primarily active at edge sites with unsaturated molybdenum sites or terminal disulfides. To improve the activity at a low loading density, two molybdenum sulfide clusters, [Mo_3_S_4_]^4+^ and [Mo_3_S_13_]^2–^, were investigated. The Mo_3_S_*x*_ molecular catalysts were heterogenized on Sb_2_Se_3_ with a simple soaking treatment, resulting in a thin catalyst layer of only a few nanometers that gave up to 20 mA cm^–2^ under one sun illumination. Both [Mo_3_S_4_]^4+^ and [Mo_3_S_13_]^2–^ exhibit catalytic activities on Sb_2_Se_3_, with [Mo_3_S_13_]^2–^ emerging as the superior catalyst, demonstrating enhanced photovoltage and an average faradaic efficiency of 100% for hydrogen evolution. This superiority is attributed to the effective loading and higher catalytic activity of [Mo_3_S_13_]^2–^ on the Sb_2_Se_3_ surface, validated by X-ray photoelectron and Raman spectroscopy.

Rising temperatures and climate variations strain natural and human systems, while an increasing energy gap, heightened by geopolitical tensions, prompts a global shift toward greener energy;^1,2^ however, the limited adoption of renewable sources, particularly in hydrogen production, underscores the need to explore alternative methods like emerging photoelectrochemical water-splitting technologies.^3,4^ Antimony selenide (Sb_2_Se_3_) has garnered attention for solar water splitting, due to its promising characteristics, including a high absorption coefficient (>10^5^ cm^–1^), photostability, and cost-effective obtainment and synthesis.^5−10^ This material shows excellent performance and stability when paired with suitable catalysts. Therefore, the search for active, easy-to-prepare, and versatile catalysts is an essential factor for the long-term success of this material. While platinum is the most commonly used catalyst for the hydrogen evolution reaction (HER) due to its high catalytic activity and minimal overpotential, its scarcity and high cost hinder large-scale deployment. As a non-noble-metal substitute, nickel and nickel alloy catalysts offer competitiveness but are typically limited to alkaline media due to corrosion in acidic environments.^11,12^ In contrast, molybdenum sulfide (MoS_*x*_) stands out for its excellent stability over a wide pH range,^13,14^ making it a promising HER catalyst for Sb_2_Se_3_ and other semiconductor materials such as Cu_2_O and GaInP.^15−19^ This catalyst can facilitate reactions in highly acidic conditions (pH 0–1) without protective overlayers such as TiO_2_.^20,21^

Various preparation methods have been employed to maximize the density of the reactive sites of the MoS_*x*_ catalyst. One promising category of these MoS_*x*_ catalysts is molybdenum sulfide clusters, which, unlike the electrochemically inert basal planes observed in MoS_2_, have maximized catalytic activity per molybdenum ion with an increased number of active sites for a given geometric surface area.^22^ Furthermore, it has been observed that the efficiency of the photoabsorber is hindered by the thicker layers of MoS_2_. However, a thin layer of the molybdenum clusters could fulfill the catalytic requirements, thereby removing any insulation effects from thicker catalyst layers.^14^

Herein, we report the suitability of versatile and easy-to-deposit catalysts directly on Sb_2_Se_3_, which include molybdenum sulfide clusters in different configurations, namely [Mo_3_S_4_(H_2_O)_9_]Cl_4_ ([Mo_3_S_4_]^4+^) and (NH_4_)_2_[Mo_3_S_13_]·2H_2_O ([Mo_3_S_13_]^2–^). These earth-abundant catalysts can provide up to 20 mA cm^–2^ at −0.3 V versus reversible hydrogen electrode (RHE) with only a few nanometers thick layers, matching the photocurrents obtained with heterogeneous cocatalysts such as platinum.^23^ The easy deposition and simplicity of these molecular catalysts make them excellent candidates for translation to other systems such as photocatalytic particles. The stability and loading of these catalysts have been studied by using XPS and Raman.

The Sb_2_Se_3_ thin films in Figure 1a form the basis for most of the devices in this study. The films are soaked in either [Mo_3_S_4_(H_2_O)_9_]Cl_4_ ([Mo_3_S_4_]^4+^) or (NH_4_)_2_[Mo_3_S_13_]·2 H_2_O ([Mo_3_S_13_]^2–^) solution for 12 h to deposit the hydrogen evolution reaction (HER) catalyst. Finally, the films are annealed at 120 °C to improve catalyst adhesion without altering molecular integrity. This was confirmed by Raman measurements which showed identical peaks before and after annealing. The [Mo_3_S_4_]^4+^ illustrated in Figure 1b is synthesized from the reaction of ammonium tetrathiomolybdate with sodium borohydride and HCl in air.^24,25^ Crystals suitable for single-crystal X-ray diffraction (XRD) were obtained after anion exchange with p-toluenesulfonic acid. The [Mo_3_S_13_]^2–^ shown in Figure 1c was synthesized according to procedures developed by Streb and co-workers,^26^ starting from ammonium heptamolybdate. A reaction with elemental sulfur and ammonium sulfide over 4 days yields the [Mo_3_S_13_]^2–^ as dark red crystals suitable for X-ray crystallography. Mimicking the MoS_2_ catalytically active edge sites,^27,28^ molybdenum sulfide clusters have a maximum dimension of approximately 0.7 nm with a high ratio of active sites to nonactive ones (e.g., the basal plane in MoS_2_); therefore, more active species and thus catalytically active sites can be packed on the photoabsorber surface, increasing reactivity with a thinly deposited catalyst layer ranging between 5–30 nm, as observed from the profilometer measurements reported in Table S1.

**Figure 1:**
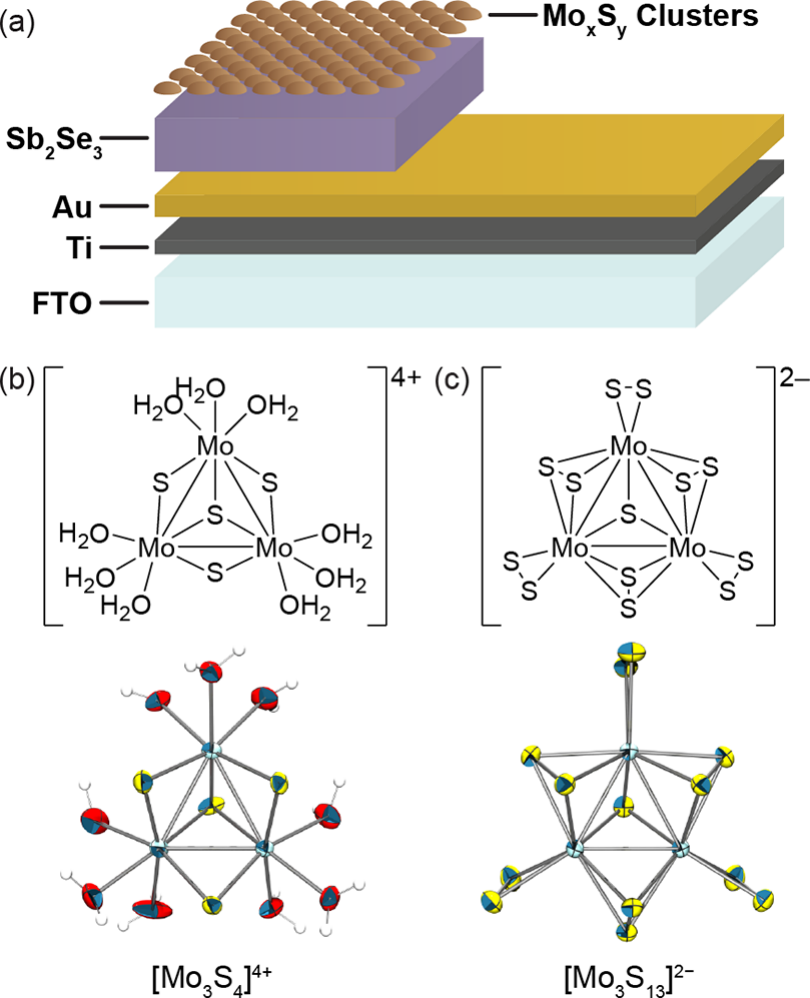
(a) Typical stack of Sb_2_Se_3_ samples with a catalyst consisting of an FTO/Ti/Au/Sb_2_Se_3_/Mo_3_S_*x*_ configuration. Molecular structure and ellipsoid displacement plots of (b) [Mo_3_S_4_]^4+^ and (c) [Mo_3_S_13_]^2–^. Ellipsoids represent 50% probability. Counterions Cl^–^ for (b), NH_4_^+^ for (c) and solvent molecules are omitted for clarity.

First, to investigate each catalyst’s activity in the dark, cyclic voltammetry (CV) cycled from 0.6 to −1.3 V versus RHE for 10 cycles in 1 M H_2_SO_4_ electrolyte (pH 0) was conducted for each sample. Figure S1 displays the CV, revealing that [Mo_3_S_13_]^2–^ exhibits a lower overpotential (approximately 300 mV less) compared to [Mo_3_S_4_]^4+^ and maintains greater stability in 1 M H_2_SO_4_ electrolyte. Tafel plots of the two catalysts are given in Figure S1b. Typically, similar slopes among a family of catalysts in a Tafel plot imply a shared mechanism. However, the observed Tafel slopes for the two molybdenum sulfur clusters [Mo_3_S_13_]^2–^ (62 mV/dec) and [Mo_3_S_4_]^4+^ (107 mV/dec) are distinct and indicate different mechanisms for the catalytic reaction.

As observed in the literature, the concentration of the catalyst solution, and therefore the catalyst loading, influences the performance of the devices.^27^ To determine the optimum concentration of each catalyst, three different catalyst concentrations were tested. The concentration of the catalyst solution was optimized at 2 mM based on measurements observed in Figure S2. Devices at this concentration outperformed others, particularly between −0.3 and −0.1 V vs RHE in [Mo_3_S_4_]^4+^ and between −0.2 and 0.0 V vs RHE in [Mo_3_S_13_]^2–^. Meanwhile at 3 mM, increased dark current was observed for both catalysts in the range of −0.3 to −0.25 V vs RHE. While these results were reproducible, sample-to-sample variation could account for the slight differences observed. Subsequently, the [Mo_3_S_4_]^4+^ and [Mo_3_S_13_]^2–^ catalysts on top of the Sb_2_Se_3_ photoabsorber were measured in 1 M H_2_SO_4_ (pH 0) under 1 sun illumination, between 0.1 to −0.3 V versus RHE. As observed in Figure 2a, the [Mo_3_S_4_]^4+^ and [Mo_3_S_13_]^2–^ catalysts produced 16.0 mA cm^–2^ and 17.5 mA cm^–2^ at −0.2 V versus RHE, respectively. With an onset potential of 0.05 V versus RHE, [Mo_3_S_13_]^2–^ has a better overall performance than [Mo_3_S_4_]^4+^ with a −0.02 V versus RHE onset. This difference is attributed to the different overpotentials of the two catalysts (Figure S1). This leads to a shift of the JV curve of the [Mo_3_S_4_]^4+^ to more negative potentials, while the [Mo_3_S_13_]^2–^ catalyst device exhibits a photocurrent approximately 2.5 mA cm^–2^ higher at −0.2 V vs RHE compared to the [Mo_3_S_4_]^4+^ catalyst device, as indicated by their J-V curves. Figure 2b illustrates that the [Mo_3_S_13_]^2–^ catalyst has a higher incident photon to current conversion efficiency (IPCE) between 400–850 nm at a similar applied potential, indicating less surface recombination and more efficient charge collection compared to [Mo_3_S_4_]^4+^, which suffers from slower charge transfer due to higher overpotential. Both catalysts have similar IPCE in the 850–1100 nm range, as differences in the extinction coefficients of Sb_2_Se_3_ affect photon absorption and charge carrier generation. In the 400–850 nm range, a high charge density near the semiconductor-electrolyte interface enhances the [Mo_3_S_13_]^2–^ catalyst’s performance. In contrast, the 850–1100 nm range sees charge generation in the semiconductor bulk, resulting in similar IPCE for both. The higher overpotential for [Mo_3_S_4_]^4+^ shown in Figure S1 underscores the [Mo_3_S_13_]^2–^ catalyst’s efficiency under conditions of high charge density at the electrolyte interface. The integrated currents in Figure S3 are slightly higher than the values observed in the CV measurements above. The slight discrepancy in values can be attributed to the light intensity dependence of the Sb_2_Se_3_ devices, as shown in previous studies.^29^ Furthermore, the activity of both catalysts was investigated across various pH levels. As shown in Figure S4, both catalysts exhibit a similar trend, performing best at pH 0, although they remain effective at more basic pH levels, as well. This study highlights the flexibility and adaptability of these catalysts in diverse pH environments; however, stability remains limited on FTO.

**Figure 2:**
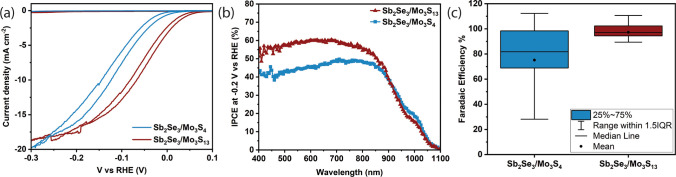
(a) Cyclic voltammetry measurements versus RHE in 1 M H_2_SO_4_ under simulated 1 sun illumination (b) IPCE measurements in 1 M H_2_SO_4_ at −0.2 V versus RHE under 1% sun-white light bias (c) Faradaic efficiency of [Mo_3_S_4_]^4*+*^ and [Mo_3_S_13_]^2–^ measured at −0.2 V versus RHE under illumination by a white LED light (∼1 sun).

As observed in Figure 2c, high faradaic efficiencies (FE) of up to 100% for the HER were obtained for both samples, as quantified with gas chromatography. [Mo_3_S_13_]^2–^ showed a FE of 103 ± 7% (Table S2) after 10 min of 1 sun illumination at an applied potential of −0.2 V versus RHE. Under the same conditions, [Mo_3_S_4_]^4+^ showed varying values with an average FE of 88 ± 17% (Table S3). The larger error for the GC measurements with [Mo_3_S_4_]^4+^ likely results from the lower photocurrent and, therefore, the longer time for the mass transport of hydrogen from the solution to the headspace, where it can be measured by GC.

The lower FE can also be attributed to the lower stability and faster degradation of the [Mo_3_S_4_]^4+^ species. On the other hand, some of the [Mo_3_S_13_]^2–^ samples were stable for over 1.5 h under harsh working conditions with high FE. This can be observed in Table S4, where FE measurements at different times are presented. CV measurements of the bare Sb_2_Se_3_ photocathode without catalysts (Figure S5a) were measured under illumination with light chopping, which showed negligible dark currents and no photocurrent. [Mo_3_S_4_]^4+^ catalyst, which is insoluble in pure H_2_O (in contrast to [Mo_3_S_13_]^2–^), was dissolved in 1 M HCl. To test the effects of the HCl solution, a bare Sb_2_Se_3_ was soaked in HCl which showed performance similar to that of the bare Sb_2_Se_3_ device (Figure S5a). The only observable difference is that the oxidation and reduction peaks seen in bare Sb_2_Se_3_ are considerably reduced in magnitude, implying that the HCl may have an etching property, removing oxides from the surface of the Sb_2_Se_3_ as previously studied.^29^ As Pt is the benchmark catalyst for HER, a bare Sb_2_Se_3_ film was deposited with 2 nm of Pt and measured under illumination by light chopping. As shown in Figure S4b, the dark current observed with the Pt catalyst is considerably increased compared with that of the bare Sb_2_Se_3_ alone. However, no photocurrent is observed with the Pt catalyst. (The Pt catalyst is known to be most effective on the Sb_2_Se_3_ when combined with a TiO_2_ overlayer, which was not investigated in this work.) The stability of each molybdenum sulfide cluster as a catalyst was investigated by measuring them through 60 cycles of CV at 10 mV s^–1^ between 0.1 V to −0.3 V versus RHE, corresponding to 80 min of measurement time (Figure S6). The [Mo_3_S_13_]^2–^ shows an improved onset and current in the first 5 cycles, then stabilizes for 20 cycles, and then starts to degrade slowly. Furthermore, chronoamperometric stability tests were conducted to evaluate the long-term stability of the catalysts (Figure S6c). The [Mo_3_S_13_]^2–^ catalyst exhibits a characteristic stability curve similar to that of the CV sweeps. The initial improvement in the current can be attributed to an increase in activity resulting from the partial exchange of disulfide ligands with aqua ligands. This substitution affects the kinetics of the hydrogen evolution reaction, making it more favorable. However, a complete exchange of disulfides for aqua ligands can lead to decreased activity due to the loss of these hydrogen evolution active sites leading to very high free energies of the Volmer step.^30,31^ In contrast, the [Mo_3_S_4_]^4+^ catalyst shows only degradation with time, with the film (Au, Sb_2_Se_3_ and catalyst) peeling off after approximately 2 h, rendering longer stability tests impossible. A partial peeling also occurs with the [Mo_3_S_13_]^2–^ catalyst, contributing to the decrease in current, which could imply that the catalyst is not the limiting factor in such stability tests. This catastrophic failure is likely due to aggressive bubble formation penetrating beneath the film, which must be addressed and optimized for further long-term stability studies.

To assess the potential morphological effects of molybdenum catalysts on the Sb_2_Se_3_ surface, scanning electron microscopy (SEM) images were obtained (Figure S7). Differences observed between bare Sb_2_Se_3_ and catalyst-treated samples were attributed to the etching treatment before catalyst soaking, consistent with prior studies.^29^ SEM images after PEC measurements showed no noticeable differences in the morphology of the films. As different films were measured for each image, the differences in grain size can be attributed to sample-to-sample or even region-to-region variation. Atomic force microscopy (AFM) studies (Figure S8) further confirmed no discernible changes in the sample’s surface morphology due to catalyst deposition. Ultraviolet–visible-near-infrared diffuse reflectance spectroscopy (UV–vis-NIR DRS) provided insights into the surface properties of samples pre and post catalyst deposition. Figure S9a shows similar reflectance spectra for bare Sb_2_Se_3_ and Sb_2_Se_3_ with [Mo_3_S_4_]^4+^, while Figure S9b reveals increased reflectance in the blue region for [Mo_3_S_13_]^2–^, corresponding to a faint brown color observed after [Mo_3_S_13_]^2–^ deposition. Raman measurements with 488, 532, and 785 nm excitation wavelengths were performed on bare Sb_2_Se_3_, Sb_2_Se_3_/[Mo_3_S_4_]^4+^ clusters as a catalyst, and Sb_2_Se_3_/ [Mo_3_S_13_]^2–^ (Figure 3). Raman measurements with 488 and 532 nm excitation wavelengths reveal clear patterns belonging to the [Mo_3_S_4_]^4+^ and [Mo_3_S_13_]^2–^ phases, as shown in Figures 3a and 3b, respectively. This behavior is expected, as both 488 and 532 nm lasers have penetration depths corresponding to about 50 and 100 nm, respectively. This reduces the ratio of Sb_2_Se_3_/[Mo_3_S_4_]^4+^, and Sb_2_Se_3_/[Mo_3_S_13_]^2–^ probed volumes, rendering these laser excitations more sensitive to detecting clusters on the surface of Sb_2_Se_3_. In the spectra of Sb_2_Se_3_ with [Mo_3_S_4_]^4+^ clusters, intense Raman peaks at 354, 440, 456, and 493 cm^–1^ are observed, which are not featured in the Raman spectra measured on the reference Sb_2_Se_3_ with both 488 and 532 nm excitations. These peak positions are in good agreement with the Raman peak positions of [Mo_3_S_4_]^4+^ reported in the literature.^32^ Furthermore, in the Raman spectra of Sb_2_Se_3_ with [Mo_3_S_13_]^2–^ clusters, peaks at 285, 329, 360, 385, 453, 518, and 552 cm^–1^ in addition to peaks belonging to the Sb_2_Se_3_ phase were identified. These peaks are in good agreement with the Raman peak positions of [Mo_3_S_13_]^2–^ phase reported in the literature.^33^ The comparison of Raman spectra measured with 785 nm does not reveal any significant differences in the spectral features, including intensity, width, and position of Raman peaks among the three samples. Only Raman peaks corresponding to the Sb_2_Se_3_ phase are observed at this wavelength, as shown in Figure 3c.^23^ This indicates that the presence of [Mo_3_S_4_]^4+^ and [Mo_3_S_13_]^2–^ clusters on the surface of Sb_2_Se_3_ do not induce any structural changes in the Sb_2_Se_3_ layer. This was corroborated by XRD measurements (Figure S10), showing no changes in crystal orientation before and after catalyst depositions. Additionally, observation of the Raman peaks belonging to the [Mo_3_S_4_]^4+^ and [Mo_3_S_13_]^2–^ phases are not expected for this excitation wavelength, as the probed volume of Sb_2_Se_3_ material is about 50 times higher than the probed volume of either [Mo_3_S_4_]^4+^ or [Mo_3_S_13_]^2–^. Furthermore, the stability and integrity of the molecular catalysts were confirmed by measuring Raman spectra after PEC measurements. As observed in Figure S11, the fingerprint regions for both catalysts are still visible at 532 nm after PEC measurements.

**Figure 3:**
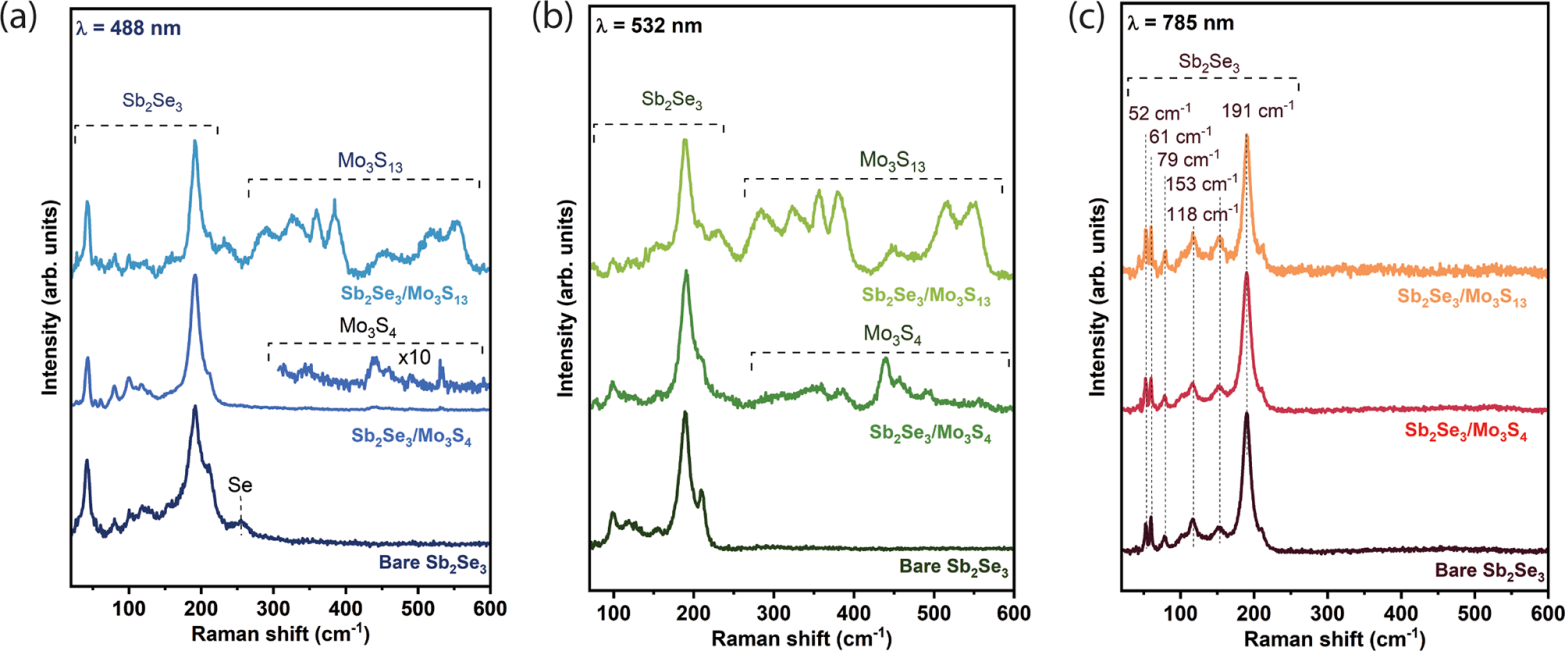
(a) Raman spectra of a typical bare Sb_2_Se_3_ sample, Sb_2_Se_3_ + [Mo_3_S_4_]^4+^ and Sb_2_Se_3_ + [Mo_3_S_13_]^2–^ at laser excitation wavelengths of (a) 488, (b) 532 nm, and (c) 785 nm.

While the mechanism of action of molybdenum sulfide catalysts is still debated, there are currently two fundamentally different theories of how HER occurs on Mo_3_S_*x*_. One is a “molybdenum based” catalysis while the other is “sulphur based”.^18^ In the molybdenum-based catalysis, unsaturated Mo sites (originating from the loss of terminal disulfides in [Mo_3_S_13_]^2–^) serve as both a redox-active element and a site for substrate binding. In this system, molybdenum hydride is generated which is then protonated to liberate H_2_.^32^ Meanwhile, in the sulfur-based system, disulfides play the dual role of proton binder and redox-active component.^35^ In both theories, a molecular system would mean that there are more active sites per unit area for H_2_ evolution as the concentration of unsaturated Mo sites in both [Mo_3_S_4_]^4+^ and [Mo_3_S_13_]^2–^ and terminal disulfides in [Mo_3_S_13_]^2–^ are higher compared to MoS_2_ thin films. Based on the electrochemical and X-ray photoelectron spectroscopy (XPS) results in this study, it is hypothesized that both H_2_ evolution mechanisms are in action in conjunction as there are no terminal disulfides in the [Mo_3_S_4_]^4+^ clusters; however, the higher activity of [Mo_3_S_13_]^2–^ is likely due to the contributions from the disulfide based hydrogen evolution leading to a better performance.^36^Figures 4a and 4b substantiate a conformal [Mo_3_S_13_]^2–^ layer with at least 6 nm thickness (which is the approximate probing depth of the Se 3d line in this measurement). This is indicated by the absence of the Sb_2_Se_3_ substrate in the Sb 3d and Se 3d spectra of both the pristine and post-PEC films. Conversely, the pristine [Mo_3_S_4_]^4+^ layer is thinner (and/or not conformal) and appears to decrease further after PEC measurements, as evidenced by the visible Sb 3d and Se 3d signals. These findings suggest a homogeneous deposition of [Mo_3_S_13_]^2–^ across the substrate, as the absence of substrate emission implies uniform coverage. Figures 4c and 4d highlight the distinction between the two catalysts. The Mo 3d spectra indicate differing oxidation states for the [Mo_3_S_4_]^4+^ and [Mo_3_S_13_]^2–^ catalysts. The pristine [Mo_3_S_13_]^2–^ catalyst shows the presence of molybdenum in Mo^4+^ form, while the post-PEC [Mo_3_S_13_]^2–^ catalyst reveals a shift in molybdenum’s core level, indicating an oxidation state of Mo^6+^, accompanied by a MoO_3_ peak. This implies the loss of some disulfides, exposing unsaturated Mo sites and enabling molybdenum-based catalysis in conjunction with the sulfur-based catalysis. Meanwhile, the S 2p spectrum of [Mo_3_S_13_]^2–^ initially shows a high concentration of disulfides (S_2_^2–^), which decreases slightly post-PEC measurements. Conversely, the Mo 3d signals for [Mo_3_S_4_]^4+^ remain unchanged but decrease in intensity post-PEC measurements. The [Mo_3_S_4_]^4+^ S 2p peaks represent only sulfide bonds (S^2–^), diminishing in intensity post-PEC measurements. NAPXPS measurements in Figure S12 illustrate a similar trend but must be interpreted with caution due to the high signal-to-noise ratio. They indicate that more catalysts could be loaded on the surface of Sb_2_Se_3_ in the case of [Mo_3_S_13_]^2–^ compared to that of [Mo_3_S_4_]^4+^ during the same soaking time and concentration. This was based on the smaller Se 3s substrate peak and more prominent Mo and S peaks for [Mo_3_S_13_]^2–^. The Se 3s substrate peak remained constant during exposure to water vapor, indicating stability. This effect was corroborated by Figure S12c and d, where Se 3d peaks were observed for [Mo_3_S_4_]^4+^ but not for [Mo_3_S_13_]^2–^. Furthermore, [Mo_3_S_13_]^2–^ clusters initially displayed the expected stoichiometry, but this changed under operation, as evidenced in Figure S12b. The spectra indicated that the coverage of [Mo_3_S_13_]^2–^ was initially very high, but after CV cycles, the Mo coverage dropped but did not entirely disappear. After CV measurements at different potentials, a chemical shift toward higher binding energy for Mo 3d was observed.

**Figure 4:**
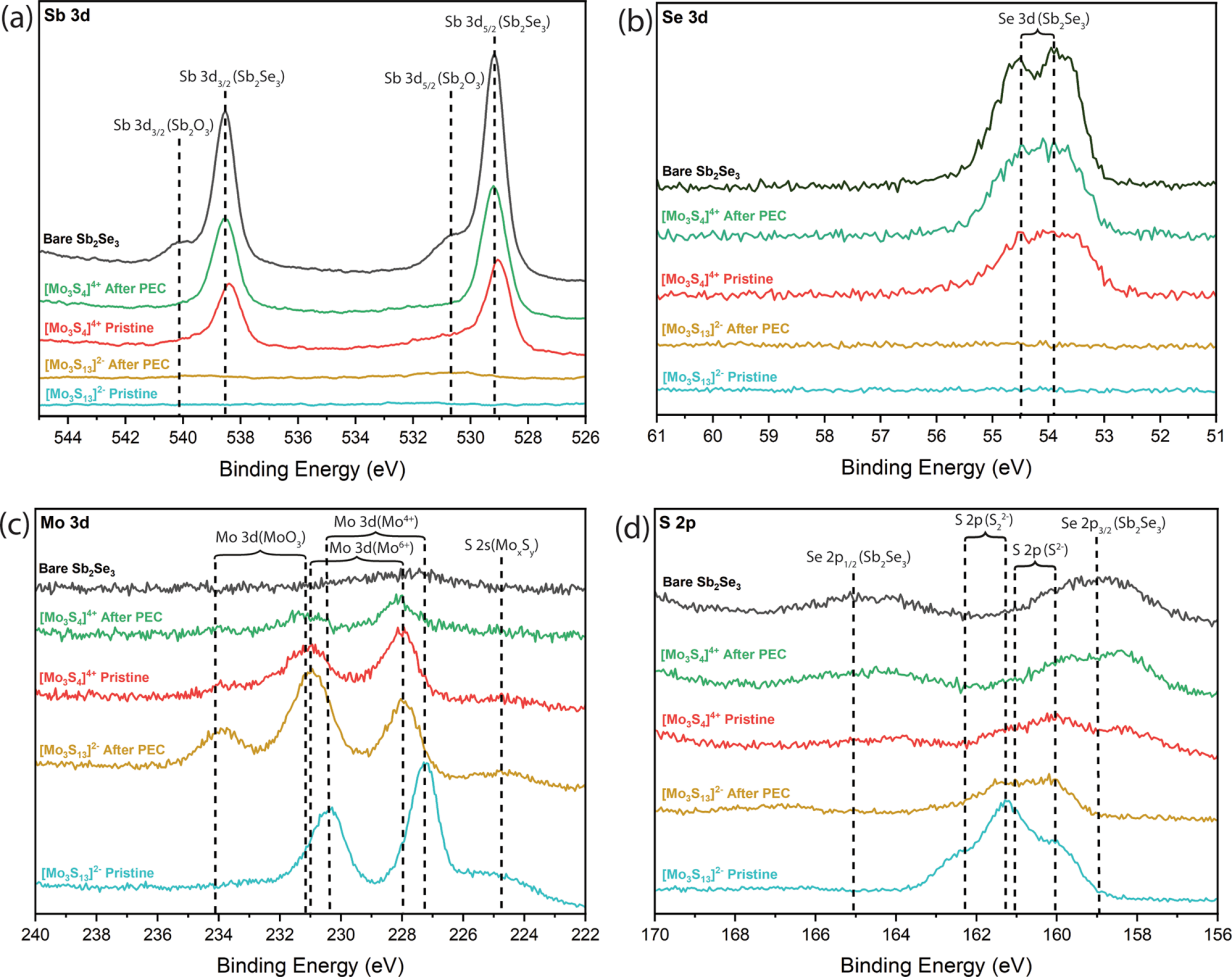
XPS spectra (a) Sb 3d (b) Se 3d (c) Mo 3d and (d) S 2p for bare and catalyzed samples measured before and after photoelectrocatalysis. Dashed reference lines are extracted from the NIST database.^34^

In summary, two molybdenum sulfide cluster species, [Mo_3_S_4_]^4+^ and [Mo_3_S_13_]^2–^, were thoroughly examined as cocatalysts on Sb_2_Se_3_ for photoelectrochemical hydrogen evolution. These clusters piqued interest due to their augmented active sites in comparison to MoS_2_ which possesses a catalytically inert basal plane. Throughout this study, it was discovered that [Mo_3_S_13_]^2–^ excelled as a catalyst when a thin layer was deposited on Sb_2_Se_3_. Remarkably, even a few tens of nanometers of catalyst deposited by soaking exhibited remarkable stability, as observed via XPS after extended use. Paired with Sb_2_Se_3_, this catalyst achieved up to 100% faradaic efficiency and a current density of 17.5 mA cm^–2^ at −0.2 V versus RHE. With additional refinement and the application of previously established treatments such as AgNO_3_ treatment and sulfurization, this catalyst, when applied to Sb_2_Se_3_, can potentially demonstrate exceptional performance and improved stability.

## Supporting Information Available

Experimental methods and materials; additional electrochemical measurements, overpotential, Tafel slopes, stability and concentration optimization, integrated currents, pH studies, control experiments; gas chromatography (GC) and Faradaic efficiencies, calibration curve and data; morphological characterization methods, scattering electron microscopy (SEM) and atomic force microscopy (AFM); Ultraviolet–visible-near-infrared diffuse reflectance spectroscopy (UV–vis-NIR DRS); near ambient pressure X-ray photoelectron spectroscopy (NAPXPS); Raman spectroscopy; single crystal and thin film X-ray diffraction (XRD) (PDF)
CIF files for compounds in Figures S14 and S15 (ZIP)

## References

[ref1] Climate Change 2022: Impacts, Adaptation and Vulnerability. https://www.ipcc.ch/report/ar6/wg2/ (accessed 2023-09-19).

[ref2] Odenweller A.; Ueckerdt F.; Nemet G. F.; Jensterle M.; Luderer G. Probabilistic Feasibility Space of Scaling up Green Hydrogen Supply. Nat. Energy 2022, 7 (9), 854–865. 10.1038/s41560-022-01097-4.

[ref3] Schneidewind J. How Much Technological Progress Is Needed to Make Solar Hydrogen Cost-Competitive?. Adv. Energy Mater. 2022, 12 (18), 2200342 10.1002/aenm.202200342.

[ref4] Zhang X.; Schwarze M.; Schomäcker R.; van de Krol R.; Abdi F. F. Life Cycle Net Energy Assessment of Sustainable H2 Production and Hydrogenation of Chemicals in a Coupled Photoelectrochemical Device. Nat. Commun. 2023, 14 (1), 991 10.1038/s41467-023-36574-1. 36813780 PMC9947173

[ref5] Yang W.; Kim J. H.; Hutter O. S.; Phillips L. J.; Tan J.; Park J.; Lee H.; Major J. D.; Lee J. S.; Moon J. Benchmark Performance of Low-Cost Sb2Se3 Photocathodes for Unassisted Solar Overall Water Splitting. Nat. Commun. 2020, 11 (1), 861 10.1038/s41467-020-14704-3. 32054858 PMC7018841

[ref6] Chen S.; Liu T.; Zheng Z.; Ishaq M.; Liang G.; Fan P.; Chen T.; Tang J. Recent Progress and Perspectives on Sb2Se3-Based Photocathodes for Solar Hydrogen Production via Photoelectrochemical Water Splitting. Journal of Energy Chemistry 2022, 67, 508–523. 10.1016/j.jechem.2021.08.062.

[ref7] Wijesinghe U.; Longo G.; Hutter O. S. Defect Engineering in Antimony Selenide Thin Film Solar Cells. Energy Advances 2022, 2, 12–33. 10.1039/d2ya00232a.

[ref8] Li Z.; Liang X.; Li G.; Liu H.; Zhang H.; Guo J.; Chen J.; Shen K.; San X.; Yu W.; Schropp R. E. I.; Mai Y. 9.2%-Efficient Core-Shell Structured Antimony Selenide Nanorod Array Solar Cells. Nat. Commun. 2019, 10 (1), 125 10.1038/s41467-018-07903-6. 30631064 PMC6328536

[ref9] Luo Y.; Chen G.; Chen S.; Ahmad N.; Azam M.; Zheng Z.; Su Z.; Cathelinaud M.; Ma H.; Chen Z.; Fan P.; Zhang X.; Liang G. Carrier Transport Enhancement Mechanism in Highly Efficient Antimony Selenide Thin-Film Solar Cell. Adv. Funct Mater. 2023, 33 (14), 2213941 10.1002/adfm.202213941.

[ref10] de Araújo M. A.; Costa M. B.; Mascaro L. H. Improved Photoelectrochemical Hydrogen Gas Generation on Sb 2 S 3 Films Modified with an Earth-Abundant MoS x Co-Catalyst. ACS Appl. Energy Mater. 2022, 5 (1), 1010–1022. 10.1021/acsaem.1c03374.

[ref11] Huo L.; Jin C.; Jiang K.; Bao Q.; Hu Z.; Chu J. Applications of Nickel-Based Electrocatalysts for Hydrogen Evolution Reaction. Advanced Energy and Sustainability Research 2022, 3, 2100189 10.1002/aesr.202100189.

[ref12] Liu X.; Ni K.; Wen B.; Guo R.; Niu C.; Meng J.; Li Q.; Wu P.; Zhu Y.; Wu X.; Mai L. Deep Reconstruction of Nickel-Based Precatalysts for Water Oxidation Catalysis. ACS Energy Lett. 2019, 4 (11), 2585–2592. 10.1021/acsenergylett.9b01922.

[ref13] Zheng H.-L.; Zhao J.-Q.; Zhang J.; Lin Q. Acid–Base Resistant Ligand-Modified Molybdenum–Sulfur Clusters with Enhanced Photocatalytic Activity towards Hydrogen Evolution. J. Mater. Chem. A Mater. 2022, 10 (13), 7138–7145. 10.1039/D2TA00352J.

[ref14] Costa M. B.; Lucas F. W. S.; Medina M.; Mascaro L. H. All-Electrochemically Grown Sb _2_ Se _3_ /a-MoS _*x*_ Photocathodes for Hydrogen Production: The Effect of the MoS _*x*_ Layer on the Surface Recombination and Photocorrosion of Sb _2_ Se < sub > 3<. ACS Appl. Energy Mater. 2020, 3 (10), 9799–9808. 10.1021/acsaem.0c01413.

[ref15] Ding Q.; Song B.; Xu P.; Jin S. Efficient Electrocatalytic and Photoelectrochemical Hydrogen Generation Using MoS2 and Related Compounds. Chem. 2016, 1 (5), 699–726. 10.1016/j.chempr.2016.10.007.

[ref16] Britto R. J.; Young J. L.; Yang Y.; Steiner M. A.; LaFehr D. T.; Friedman D. J.; Beard M.; Deutsch T. G.; Jaramillo T. F. Interfacial Engineering of Gallium Indium Phosphide Photoelectrodes for Hydrogen Evolution with Precious Metal and Non-Precious Metal Based Catalysts. J. Mater. Chem. A Mater. 2019, 7 (28), 16821–16832. 10.1039/C9TA05247J.

[ref17] Morales-Guio C. G.; Tilley S. D.; Vrubel H.; Grätzel M.; Hu X. Hydrogen Evolution from a Copper(I) Oxide Photocathode Coated with an Amorphous Molybdenum Sulphide Catalyst. Nat. Commun. 2014, 5 (1), 3059 10.1038/ncomms4059. 24402352

[ref18] Grutza M.-L.; Rajagopal A.; Streb C.; Kurz P. Hydrogen Evolution Catalysis by Molybdenum Sulfides (MoSx): Are Thiomolybdate Clusters like [Mo3S13]2- Suitable Active Site Models?. Sustain Energy Fuels 2018, 2 (9), 1893–1904. 10.1039/C8SE00155C.

[ref19] Laursen A. B.; Kegnæs S.; Dahl S.; Chorkendorff I. Molybdenum Sulfides - Efficient and Viable Materials for Electro - And Photoelectrocatalytic Hydrogen Evolution. Energy and Environmental Science 2012, 5, 5577 10.1039/c2ee02618j.

[ref20] Prabhakar R. R.; Septina W.; Siol S.; Moehl T.; Wick-Joliat R.; Tilley S. D. Photocorrosion-Resistant Sb2Se3 Photocathodes with Earth Abundant MoS: X Hydrogen Evolution Catalyst. J. Mater. Chem. A Mater. 2017, 5 (44), 23139–23145. 10.1039/C7TA08993G.

[ref21] Gu J.; Aguiar J. A.; Ferrere S.; Steirer K. X.; Yan Y.; Xiao C.; Young J. L.; Al-Jassim M.; Neale N. R.; Turner J. A. A Graded Catalytic-Protective Layer for an Efficient and Stable Water-Splitting Photocathode. Nat. Energy 2017, 2 (2), 16192 10.1038/nenergy.2016.192.

[ref22] Batool S.; Nandan S. P.; Myakala S. N.; Rajagopal A.; Schubert J. S.; Ayala P.; Naghdi S.; Saito H.; Bernardi J.; Streb C.; Cherevan A.; Eder D. Surface Anchoring and Active Sites of [Mo _3_ S _13_ ] ^2–^ Clusters as Co-Catalysts for Photocatalytic Hydrogen Evolution. ACS Catal. 2022, 12 (11), 6641–6650. 10.1021/acscatal.2c00972. 35692252 PMC9171716

[ref23] Adams P.; Schnyder R.; Moehl T.; Bühler J.; Alvarez A. L.; Dimitrievska M.; McKenna K.; Yang W.; Tilley S. D. Post-Synthetic Silver Ion and Sulfurization Treatment for Enhanced Performance in Sb 2 Se 3 Water Splitting Photocathodes. Adv. Funct Mater. 2023, 2310596 10.1002/adfm.202310596.

[ref24] Shibahara T.; Yamasaki M.; Sakane G.; Minami K.; Yabuki T.; Ichimura A. Syntheses and Electrochemistry of Incomplete Cubane-Type Clusters with M3S4 Cores (M = Molybdenum, Tungsten). X-Ray Structures of [W3S4(H2O)9](CH3C6H4SO3)4.Cntdot.9H2O, Na2[W3S4(Hnta)3].Cntdot.5H2O, and (BpyH)5[W3S4(NCS)9].Cntdot.3H2O. Inorg. Chem. 1992, 31 (4), 640–647. 10.1021/ic00030a022.

[ref25] Inorganic Syntheses; Coucouvanis D.,, Ed.; Wiley, 2002; Vol. 33. 10.1002/0471224502.

[ref26] Dave M.; Rajagopal A.; Damm-Ruttensperger M.; Schwarz B.; Nägele F.; Daccache L.; Fantauzzi D.; Jacob T.; Streb C. Understanding Homogeneous Hydrogen Evolution Reactivity and Deactivation Pathways of Molecular Molybdenum Sulfide Catalysts. Sustain Energy Fuels 2018, 2 (5), 1020–1026. 10.1039/C7SE00599G.

[ref27] Kibsgaard J.; Jaramillo T. F.; Besenbacher F. Building an Appropriate Active-Site Motif into a Hydrogen-Evolution Catalyst with Thiomolybdate [Mo3S13]2- Clusters. Nat. Chem. 2014, 6 (3), 248–253. 10.1038/nchem.1853. 24557141

[ref28] Huang Z.; Luo W.; Ma L.; Yu M.; Ren X.; He M.; Polen S.; Click K.; Garrett B.; Lu J.; Amine K.; Hadad C.; Chen W.; Asthagiri A.; Wu Y. Dimeric [Mo2S12]2- Cluster: A Molecular Analogue of MoS2 Edges for Superior Hydrogen-Evolution Electrocatalysis. Angewandte Chemie - International Edition 2015, 54 (50), 15181–15185. 10.1002/anie.201507529. 26482571

[ref29] Adams P.; Creazzo F.; Moehl T.; Crockett R.; Zeng P.; Novotny Z.; Luber S.; Yang W.; Tilley S. D. Solution Phase Treatments of Sb 2 Se 3 Heterojunction Photocathodes for Improved Water Splitting Performance. J. Mater. Chem. A Mater. 2023, 11 (15), 8277–8284. 10.1039/D3TA00554B. 37066134 PMC10088359

[ref30] Dave M.; Rajagopal A.; Damm-Ruttensperger M.; Schwarz B.; Nägele F.; Daccache L.; Fantauzzi D.; Jacob T.; Streb C. Understanding Homogeneous Hydrogen Evolution Reactivity and Deactivation Pathways of Molecular Molybdenum Sulfide Catalysts. Sustain Energy Fuels 2018, 2 (5), 1020–1026. 10.1039/C7SE00599G.

[ref31] Escalera-López D.; Iffelsberger C.; Zlatar M.; Novčić K.; Maselj N.; Van Pham C.; Jovanovič P.; Hodnik N.; Thiele S.; Pumera M.; Cherevko S. Allotrope-Dependent Activity-Stability Relationships of Molybdenum Sulfide Hydrogen Evolution Electrocatalysts. Nature Communications 2024 15:1 2024, 15 (1), 1–13. 10.1038/s41467-024-47524-w. PMC11058198 38684654

[ref32] Tran P. D.; Tran T. V.; Orio M.; Torelli S.; Truong Q. D.; Nayuki K.; Sasaki Y.; Chiam S. Y.; Yi R.; Honma I.; Barber J.; Artero V. Coordination Polymer Structure and Revisited Hydrogen Evolution Catalytic Mechanism for Amorphous Molybdenum Sulfide. Nat. Mater. 2016, 15 (6), 640–646. 10.1038/nmat4588. 26974410 PMC5495159

[ref33] Yuan M.; Yao H.; Xie L.; Liu X.; Wang H.; Islam S. M.; Shi K.; Yu Z.; Sun G.; Li H.; Ma S.; Kanatzidis M. G. Polypyrrole–Mo _3_ S _13_ : An Efficient Sorbent for the Capture of Hg ^2+^ and Highly Selective Extraction of Ag ^+^ over Cu ^2+^. J. Am. Chem. Soc. 2020, 142 (3), 1574–1583. 10.1021/jacs.9b12196. 31855420

[ref34] Naumkin A. V.; Kraut-Vass A.; Gaarenstroom S. W.; Powell C. J. NIST X-ray Photoelectron Spectroscopy Database. https://srdata.nist.gov/xps/ (accessed 2024-05-30).

[ref35] Lassalle-Kaiser B.; Merki D.; Vrubel H.; Gul S.; Yachandra V. K.; Hu X.; Yano J. Evidence from in Situ X-Ray Absorption Spectroscopy for the Involvement of Terminal Disulfide in the Reduction of Protons by an Amorphous Molybdenum Sulfide Electrocatalyst. J. Am. Chem. Soc. 2015, 137 (1), 314–321. 10.1021/ja510328m. 25427231 PMC4304453

[ref36] Batool S.; Langer M.; Myakala S. N.; Heiland M.; Eder D.; Streb C.; Cherevan A. Thiomolybdate Clusters: From Homogeneous Catalysis to Heterogenization and Active Sites. Adv. Mater. 2023, 36 (7), 2305730 10.1002/adma.202305730. PMC11475511 37899494

