# Supplementary material for: Direct Anchoring of Molybdenum Sulfide Molecular Catalysts on Antimony Selenide Photocathodes for Solar Hydrogen Production
Source: ACS Energy Lett. 2024 Jul 12;9(8):3828–34. doi: 10.1021/acsenergylett.4c01570 (PMC11320643; doi:10.1021/acsenergylett.4c01570)
Supplement: Supplementary file 1 — nz4c01570_si_001.pdf [file nz4c01570_si_001.pdf]

Supporting Information

Direct Anchoring of Molybdenum Sulfide Molecular Catalysts on Antimony Selenide Photocathodes for Solar Hydrogen Production

Pardis Adams[†], Jan Bühler[†], Iva Walz[†], Thomas Moehl[†], Helena Roithmeyer[†], Olivier Blacque[†], Nicolò Comini[‡§], J. Trey Diulus[‡§], Roger Alberto[†], Sebastian Siol[‖], Mirjana Dimitrievska[‖], Zbynek Novotny[‡§], and S. David Tilley[†]**

[†] Department of Chemistry, University of Zurich, Zurich, 8057, Switzerland

[‡] Department of Physics, University of Zurich, Zurich, 8057, Switzerland

[§] Swiss Light Source, Paul Scherrer Institute, Villigen, 5232, Switzerland

[‖] Surface Science and Coating Technologies Laboratory/ Transport at Nanoscale Interfaces Laboratory, Swiss Federal Laboratories for Materials Science and Technology (EMPA), Dübendorf, 8600, Switzerland

Corresponding Author

S. David Tilley – Department of Chemistry, University of Zurich, Zurich, 8057, Switzerland,

E-mail: david.tilley@chem.uzh.ch

Zbynek Novotny – Department of Physics, University of Zurich, Zurich, 8057, Switzerland,

E-mail: zbynek.novotny@pnnl.gov

EXPERIMENTAL METHODS

General Information

Unless otherwise stated, all chemicals were of reagent grade or higher, obtained from commercial sources and used without further purification. Solvents for reactions were of p.a. grade; H_2O was ultrapure from a Milli-Q® Direct 8 water purification system. Deuterated solvents were purchased from Sigma Aldrich.

Catalyst Synthesis

$[Mo_3S_4(H_2O)_9]Cl_4$

Prepared according to a published procedure.[1,2]

Caution: Toxic H_2S is produced during the reaction. It is recommended to use gas washing bottles containing bleach.

To a solution of $(NH_4)_2[MoS_4]$ (1.00 g, 3.84 mmol) in H_2O (35 mL) were alternatingly added (1 mL each addition) aqueous HCl (6 M, 15 mL) and aqueous $NaBH_4$ (2 M, 15 mL). The brown reaction mixture was heated to 90 °C while air was passed through a glass syringe. After 22 h, a colour change to dark green was observed, and the mixture was cooled with an ice bath. Filtration and washing of the filter cake with aqueous HCl (1 M, 10 mL) afforded a green filtrate condensed in

vacuo to roughly 5 mL. The crude product was purified over a Sephadex G-10 (10 g, bloomed in 1 M HCl, 1 M HCl as eluent) column. A green band of $[Mo_3S_3O]^{4+}$ (λ_{max} 605 nm) was eluted first before a band containing the dark green product (λ_{max} 620 nm). The product-containing fractions were diluted with H_2O (five times the original volume) and purified over a DOWEX 50WX2 (15 g, washed with 2 M HCl, 2 M as eluent) column. A light brown band of $[Mo_2O_2S_2]^{2+}$ was eluted first before a band of the dark green product. The solvent was evaporated in vacuo to afford $[Mo_3S_4(H_2O)_9]Cl_4$ (362 mg, 1.281 mmol, 39%) as a dark green powder.

FT-IR (KBr, cm^{-1}): 3390 (br, s), 3225 (s), 1622 (m), 1404 (m), 1195 (w), 960 (w), 847 (m), 806 (m), 570 (w), 493 (w).

HRMS (ESI) m/z calcd for $C_2H_6ClMo_3O_2S_4$ $[M-9\ H_2O+2\ OMe+Cl]^+$: 518.60958; found: 518.60871.

UV-Vis (1 M HCl, nm ($mol^{-1}\ dm^3\ cm^{-1}$)): λmax (ε)= 255 (7'945), 371 (3'475), 620 (172).

$(NH_4)_2[Mo_3S_{13}]\cdot 2\ H_2O$

Prepared according to a published procedure.[3]

$(NH_4)_2S_x$ (25 wt-%) solution was prepared by dissolving elemental sulphur (3.00 g) in $(NH_4)_2S$ (48 wt-%, 20 mL) and H_2O (20 mL). $(NH_4)_2[Mo_7O_{24}]\cdot 4\ H_2O$ (1.02 g, 0.825 mmol) was dissolved in

H_2O (5 mL), and aqueous $(NH_4)_2S_x$ solution (25 wt-%, 30 mL) was added. The flask was covered with a watch glass, and the reaction mixture was heated to 95 °C for 96 h without stirring. Dark red crystals were formed together with solid elemental sulphur. The solids were collected by filtration, and the filter cake was washed with H_2O (3×10 mL), EtOH (3×10 mL), CS_2 (3×10 mL, until sulfur has fully dissolved) and Et_2O (3×10 mL). The filter cake was air dried to yield $(NH_4)_2[Mo_3S_{13}]\cdot 2\ H_2O$ (1.32 g, 1.70 mmol, 88%) as dark red crystals.

FT-IR (KBr, cm^{-1}): 3437 (br, m), 3081 (m), 2926 (m), 2781 (m), 1633 (w), 1566 (w), 1399 (s), 1385 (s), 544 (s), 506 (s), 459 (w).

HRMS (ESI) m/z calcd for $H_3Mo_3S_{13}$ $[M+3\ H]^+$: 712.37607; found: 712.37391.

UV-Vis (MeOH, nm ($mol^{-1}\ dm^3\ cm^{-1}$)): λmax (ε)= 267 (38’978), 425 (4’432).

Catalyst Characterization

FT-IR spectra were recorded on a SpectrumTwo FT-IR Spectrometer (Perkin–Elmer); samples were applied as KBr pellets. **High-resolution electrospray mass spectra (HR-ESI-MS)** were recorded on a timsTOF Pro TIMS-QTOF-MS instrument (Bruker Daltonics GmbH, Bremen, Germany). The samples were dissolved in MeOH at a ca. 50 μg mL^{-1} concentration and analyzed via continuous flow injection (2 μL min^{-1}). The mass spectrometer was operated in the positive or negative electrospray ionization mode at 4'000 V (-4'000 V) capillary voltage and −500 V (500 V)

endplate offset with an N_2 nebulizer pressure of 0.4 bar and a dry gas flow of 4 L min^{-1} at 180 ˚C. Mass spectra were acquired in a mass range from m/z 50 to 2'000 at ca. 20'000 resolution (m/z 622) and at 1.0 Hz rate. The mass analyzer was calibrated between m/z 118 and 2'721 using an Agilent ESI-L low-concentration tuning mix solution (Agilent, USA) at a resolution of 20,000, giving a mass accuracy below 2 ppm. All solvents used were purchased in the best LC-MS quality. **UV-Vis** spectra were recorded on a Shimadzu UV-3600 Plus spectrophotometer.

PEC Device Synthesis

Pilkington's FTO TEC 15 substrates were first cut into 1×2.5 cm^2 pieces and then meticulously cleaned using a series of solvents: soapy distilled water, distilled water, acetone, and isopropyl alcohol (IPA). Following this cleaning process, they were dried with a nitrogen gun. Subsequently, the substrates underwent a 30 minute UV/ozone cleaning to eliminate surface contaminants. Next, a Safematic CCU-010 sputter coater was employed to apply a 10 nm thick layer of titanium (Ti) (acting as an adhesion layer) followed by a 150 nm thick layer of gold (Au) onto the FTO substrates (serving as a hole-extracting electrode, creating an ohmic contact with the photoabsorber). A three-electrode setup was utilized to carry out the electrodeposition of antimony (Sb) metal onto the FTO/Ti/Au substrates. The Sb electrodeposition solution consisted of 15 mM potassium antimony tartrate and 50 mM tartaric acid, with the pH adjusted to 1.3. An electrode potential of −0.3 V versus Ag/AgCl was applied to control the Sb thickness by monitoring the charge passed, limited

to 1.4 C cm^{-2}. Careful attention was given to ensure uniform thickness and optimal performance during deposition. Subsequently, the resulting Sb substrates underwent a selenization process using a two-zone furnace. Selenium pellets were positioned around the substrate, and the chamber was purged with argon. The temperature was gradually increased to 350 ˚C at a rate of 15 ˚C per minute and held for 40 minutes. For the reference, Sb_2Se_3/Pt samples, a 2 nm thick platinum (Pt) layer was sputtered onto the photocathode. This method creates high-quality compact thin films without requiring advanced high vacuum equipment. However, it is crucial to exercise caution to prevent minor gas leaks that could lead to forming an Sb_2O_3 layer on top of the Sb_2Se_3.

Photoelectrochemical (PEC) Characterization of Sb_2Se_3

The photoelectrochemical performance of the photocathodes was evaluated using a BioLogic SP-200 potentiostat. This assessment followed a three-electrode setup, with simulated AM 1.5G illumination provided and calibrated to 100 mW cm^{-2} (1 sun) using a silicon diode sourced from PV Measurements, Inc. The electrolyte used was a 1 M H_2SO_4 solution with a pH of 0. The three-electrode configuration consisted of an Ag/AgCl reference electrode immersed in a 3 M KCl solution, a counter electrode made of freshly cleaned Pt wire, and the photocathode serving as the working electrode. Cyclic voltammetry (CV) measurements were conducted at a scan speed of 10 mV s^{-1}. The scans were performed by sweeping from positive to negative potential and then back

to positive potential. The exact onset potential value was defined by extrapolating to zero current from the oxidative sweep at the HER peak. To define the photocathode area, epoxy (specifically Loctite 9461) was applied around an O-ring with an inner diameter of 7 mm and placed on the sample's surface. For measurements of incident photon-to-current efficiency (IPCE), a custom-built IPCE system was utilized. This system included a halogen light source, a double monochromator, and white light bias generated by an LED. IPCE measurements were also conducted within the aforementioned three-electrode configuration, maintaining a potential of –0.2 V versus RHE (Reversible Hydrogen Electrode). These measurements were performed with 5 nm wavelength intervals and a 1% white light bias.

Solution Treatments and Catalyst Deposition

In this section, all procedures were carried out following the synthesis of Sb_2Se_3 and preceding the catalyst soaking. Prior to any of these treatments, a layer of Teflon tape was applied to mask the exposed Au surface. Subsequently, the Sb_2Se_3 thin films were briefly immersed in a transparent yellow solution of $(NH_4)_2S$ (10 wt%) for 5 seconds. They were then rinsed with distilled water and dried under a nitrogen stream (N_2). This particular sequence of steps was undertaken as the initial measure before further treatments, owing to its effectiveness in enhancing device performance, as previously detailed in our earlier publication.[4] For a 1 mM $[Mo_3S_4]^{4+}$ catalyst deposition, 7.20 mg of $[Mo_3S_4(H_2O)_9]Cl_4$ were dissolved in 10 ml 1 M HCl and sonicated for 30 minutes. Similarly, for 1 mM $[Mo_3S_{13}]^{2-}$ catalyst deposition, 7.77 mg $(NH_4)_2[Mo_3S_{13}]\cdot 2\ H_2O$ were dissolved in 10 ml

distilled H_2O and sonicated for 30 minutes. Samples were placed in the catalyst solution and soaked for 12 hours at room temperature. They were then rinsed from the back and annealed at 120° C for 30 minutes. The same procedure was performed for the catalyst deposition on FTO.

Tafel Plot Analysis

The LSV measurements were conducted using an Ag/AgCl reference electrode in 1 M H_2SO_4, with a Pt counter electrode placed behind a frit to prevent oxygen entry, but still in the same electrolyte solution. The potential range applied was from 0.2 to –0.55 V vs RHE (against RHE) for $[Mo_3S_4]^{4+}$, and from 0.2 to -0.35 V vs RHE for $[Mo_3S_{13}]^{2-}$, with a scan rate of 0.5 mV/s. To stabilize the redox potential in the solution, hydrogen was bubbled electrochemically using two Pt wires connected to a second potentiostat. The Pt electrode generating oxygen was also behind a frit but remained in the same electrolyte solution. For the Tafel plot analysis, the applied potential was IR drop corrected using the formula: IR drop corrected potential = applied voltage – voltage drop, where the voltage drop was calculated by multiplying the current by the series resistance. The series resistance (30 ohms) was determined through electrochemical impedance spectroscopy at 0.2 V. A linear fit was performed for both the initial rise in current (first redox event) and the subsequent redox event leading to H_2 generation. The latter fit was in the range of 0.1 to 1 mA/cm².

GC and Faradaic Efficiency

The produced hydrogen was measured on an Inficon Fusion Micro gas chromatograph with a molecular sieve column (5Å) and a μTCD. Argon was used as the carrier gas. Before starting the measurements, the samples were placed in a one-component cell with a reference and a counter electrode. They were degassed for 30 to 40 minutes with argon, and three baseline measurements were done before each sample measurement. The operation pressure was between 1.020 and 1.030 bar and kept constant with a pressure control system. The calibration (and associated calculations) are seen and described in the supplementary information, **Figure S10** and **Table S3**.

Morphology and Crystal Characterization

Top-view scanning electron microscopy (SEM) images of the Sb_2Se_3 thin films with and without different catalysts were acquired using a Zeiss Gemini 450 SEM. X-ray diffraction (XRD) analysis utilized the Rigaku Smartlab diffractometer. Reference cards for Sb_2Se_3 and Au were obtained from the Cambridge Crystallographic Data Centre (CCDC) database. The UV-3600 Plus instrument from Shimadzu, equipped with an integrating sphere, was employed for conducting diffuse reflectance measurements (DRS). An Asylum Research AFM (MFP-3D) was used to measure the work function of the samples. The probe used for the measurement was an AC240TM-R3. For calibration of the work function of the tip, a highly ordered pyrolytic graphite (HOPG) was used, with a reported work function of ~4.6 eV.[5] To achieve a fresh HOPG surface, a piece of scotch tape was used to pull off a few top layers of the graphite, exposing a fresh, clean surface for calibration. The HOPG used was purchased from MikroMasch (Grade: ZYA). The open-source

Gwyddion software package and the Asylum Research built-in software were used to analyse the AFM pictures further. The thickness of the samples was measured using the DektakXT Bruker stylus profilometer.

Raman

Raman measurements were acquired on a WITec Alpha 300 R confocal Raman microscope in backscattering geometry. Multiwavelength excitation Raman measurements using 488, 532 and 785 nm lasers were performed on all samples. The beam was focused on the sample with a microscope objective, resulting in a diameter spot of 800 nm for 488 nm laser, 1 μm for 532 nm laser, and 1.2 μm for 785 nm laser, and reaching a radiant power of the order of 300 μW. Laser power conditions were selected based on a power study, which involved measuring Raman spectrum at the same point on the material with increasing laser power densities, starting from the lowest power available. For each laser power, the spectrum was monitored for changes in peak positions, peak widths, or the appearance of new peaks. The highest power for which no changes in these parameters were observed was taken as the optimal laser power for measurements. The backscattered light was analyzed with two spectrometers: a 300 mm lens-based spectrometer with a grating of 1800 g/mm equipped with a thermoelectrically cooled CCD for 488 and 532 nm excitation and a 400 mm lens-based spectrometer with a grating of 1200 g/mm equipped with a

cooled deep-depletion CCD for 785 nm excitation. All spectra were calibrated based on the reference Si Raman spectrum.

XPS

All XPS spectra were recorded using the endstation described in the referenced literature.[6] XPS spectra were recorded using focused, linearly polarized light 4000 eV at the Phoenix I beamline at the Swiss Light Source, with a probing depth of approximately 30 nm in liquid water. Spectra were recorded in high vacuum (HV) and at equilibrium electrolyte at a water vapor pressure of 25 mbar. Measurements were taken at three different potentials, which varied for each catalyst. P1(before onset potential), P2 (at onset potential) and P3 (after onset potential) were set to +0.22, +0.02 and –0.18 mV vs RHE for $[Mo_3S_4]^{+4}$, respectively. Moreover, P1, P2 and P3 were set to +0.05, –0.15 and –0.3 mV vs RHE for $[Mo_3S_{13}]^{2-}$, respectively. Peak fitting was performed in CasaXPS, and spectra were plotted in Origin Pro. The combined Mo 3d and S 2s region was fitted with two pairs of peaks for both Mo 3d and S2s. Two different binding configurations are further supported by the S 2p region that shows two distinct S 2p peaks. All peaks were fitted with a GL(30) function, and peak area ratio and spin-orbit splitting were constrained. The same full width at half maxima (FWHM) is used for peak doublets for both peaks.

Samples	$[Mo_3S_{13}]^{2-}$	$[Mo_3S_4]^{4+}$
**1**	30.97	6.45
**2**	15.92	5.97
**3**	30.25	6.93
**4**	30.23	5.10
**5**	33.74	6.04

Table S1 – Thickness of five different samples as measured by the profilometer (Dektac, Bruker), measuring the step difference between FTO and catalyst.

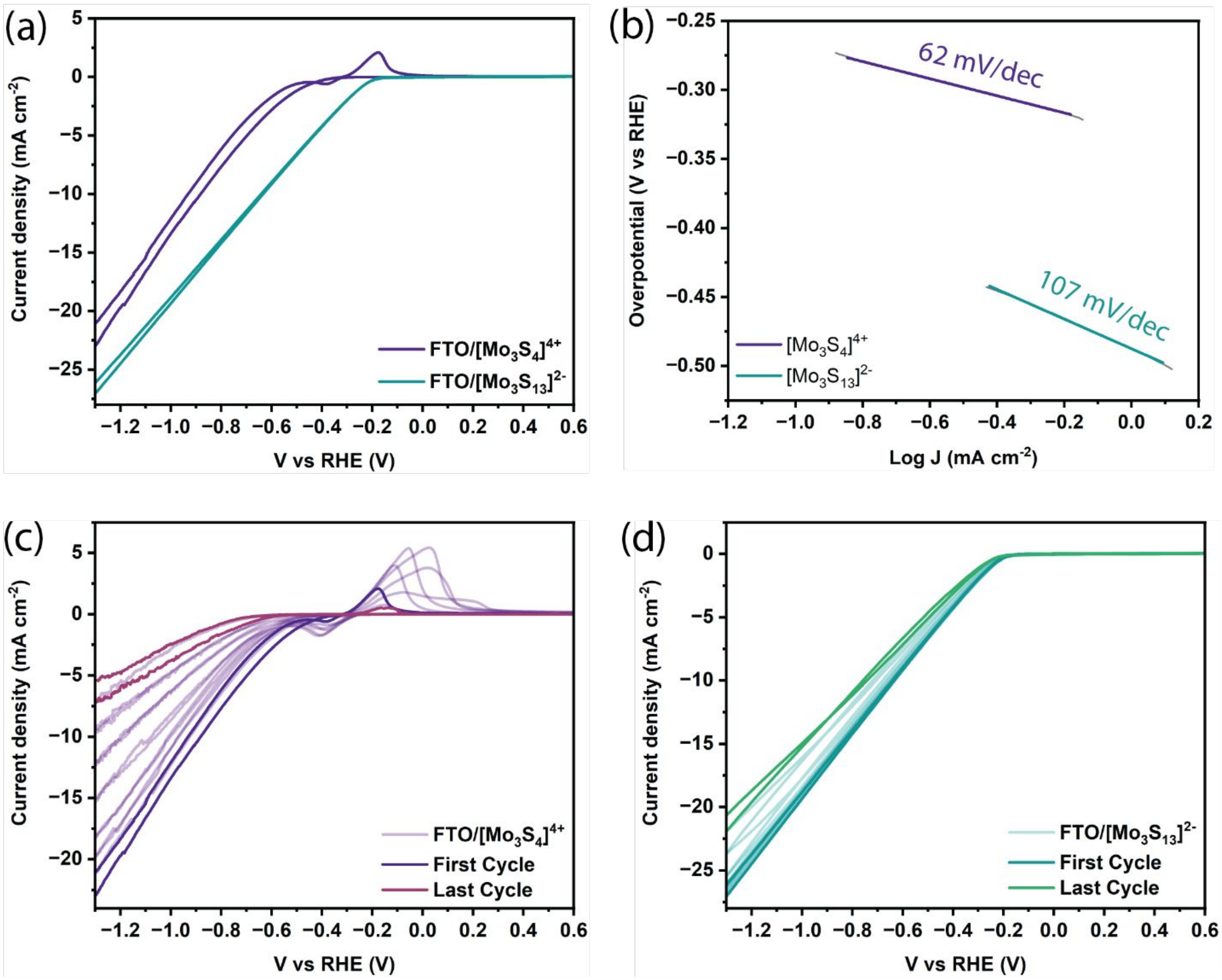

Figure S1 – (a) Dark CV measurements of $[Mo_3S_4]^{4+}$ and $[Mo_3S_{13}]^{2-}$ only on an FTO substrate (b) Tafel plots of $[Mo_3S_4]^{4+}$ and $[Mo_3S_{13}]^{2-}$an FTO substrate, IR drop corrected (c) performance of $[Mo_3S_4]^{4+}$ over 10 cycles (d) performance of $[Mo_3S_{13}]^{2-}$ over 10 cycles.

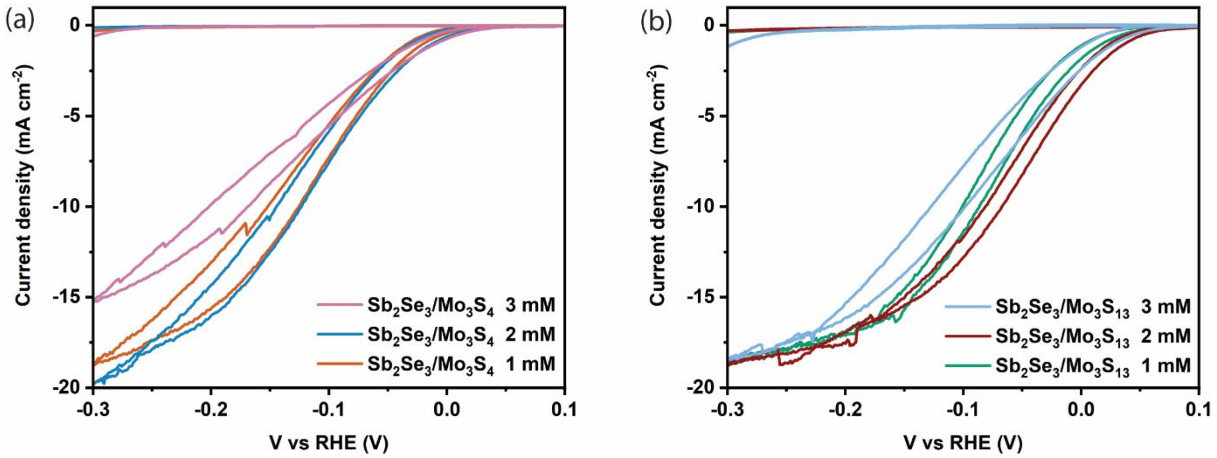

Figure S2 – (a) Cyclic voltammetry measurements of Sb_2Se_3 + $[Mo_3S_4]^{4+}$ samples at various concentrations at 1 sun illumination and in the dark (b) Cyclic voltammetry measurements of Sb_2Se_3 + $[Mo_3S_{13}]^{2-}$ samples at various concentrations at 1 sun illumination and in the dark.

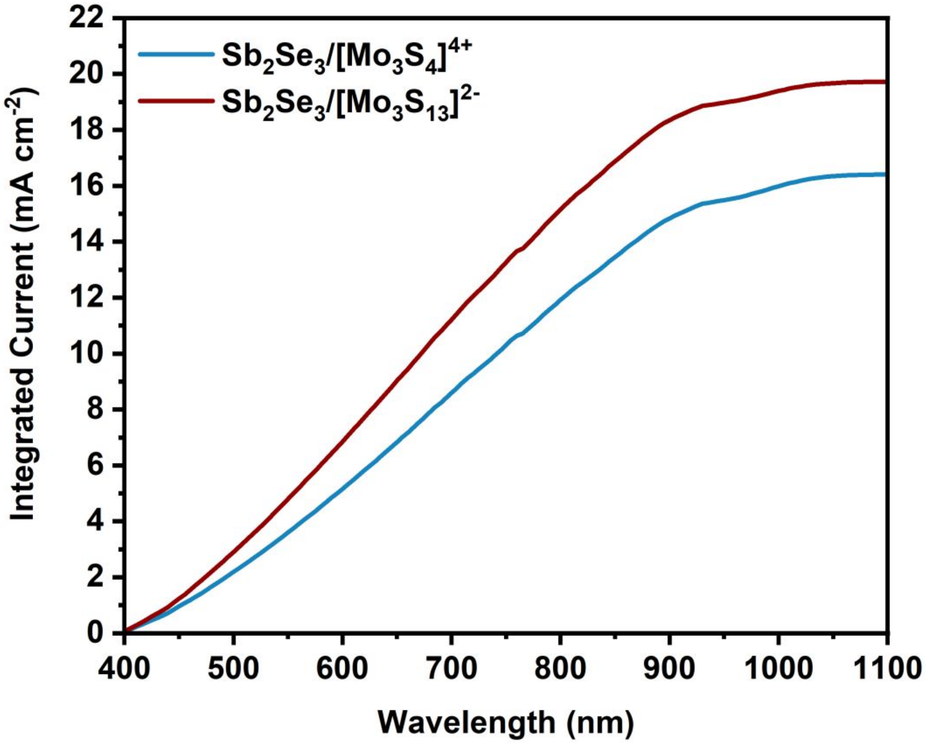

Figure S3 – Integrated photocurrents (mA cm^{-2}) based on the IPCE measurements in Fig 2b and under AM 1.5 G (100 mW cm^{-2}) simulated solar irradiation.

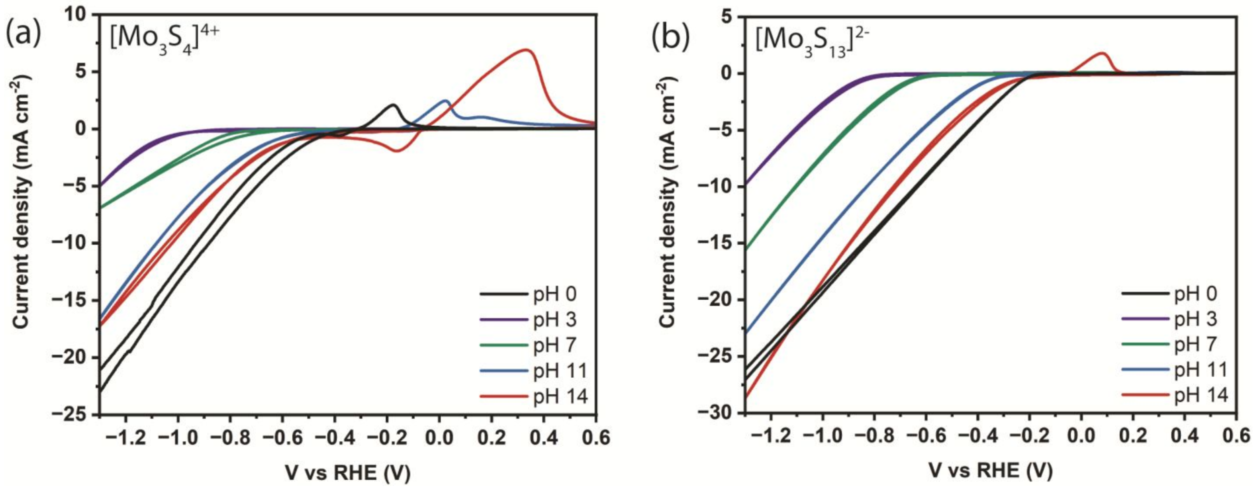

Figure S4 – (a) Cyclic voltammetry measurements of FTO + $[Mo_3S_4]^{4+}$ samples in different pH environments (b) Cyclic voltammetry measurements of FTO + $[Mo_3S_{13}]^{2-}$ samples in different pH environments.

$[Mo_3S_4]^{4+}$	S1	S2	S3	Average ± STD
H_2 (mol)	$2.13 \cdot 10^{-6}$	$1.06 \cdot 10^{-6}$	$1.43 \cdot 10^{-7}$	
Q_{el}/ C	0.403	0.219	0.0401	
FE (%)	102	93	69	88 ± 17

Table S2 – Measured and calculated values for $[Mo_3S_4]^{4+}S_4$ after applying –0.2 V versus RHE under 1 sun illumination for 10 min

$[Mo_3S_{13}]^{2-}$	S1	S2	S3	Average ± STD
H_2 (mol)	$1.61 \cdot 10^{-5}$	$2.06 \cdot 10^{-5}$	$6.08 \cdot 10^{-6}$	
Q_{el} (C)	3.20	3.60	1.15	
FE (%)	97	111	102	103 ± 7

Table S3 – Measured and calculated values for $[Mo_3S_{13}]^{2-}$ after applying –0.2 V versus RHE under 1 sun illumination for 10 min

	$[Mo_3S_{13}]^{2-}$		$[Mo_3S_4]^{4+}$	
t/min	mol H_2	FE%	mol H_2	FE%
1	$8.74 . 10^{-7}$	55	$8.14 . 10^{-8}$	9
5	$7.65 . 10^{-6}$	98	$6.29 . 10^{-7}$	21
10	$2.06 . 10^{-5}$	110	$1.06 . 10^{-6}$	93
20	$3.03 . 10^{-5}$	80	$1.39 . 10^{-6}$	112
30	$3.86 . 10^{-5}$	102	-	-
30	$3.03 . 10^{-5}$	80	-	-

Table S4 – Moles of hydrogen and faradaic efficiency of representative samples at different times (time is cumulative, e.g. $[Mo_3S_{13}]^{2-}$ the sample was measured for 96 minutes in total) at –0.2 V versus RHE under 1 sun illumination.

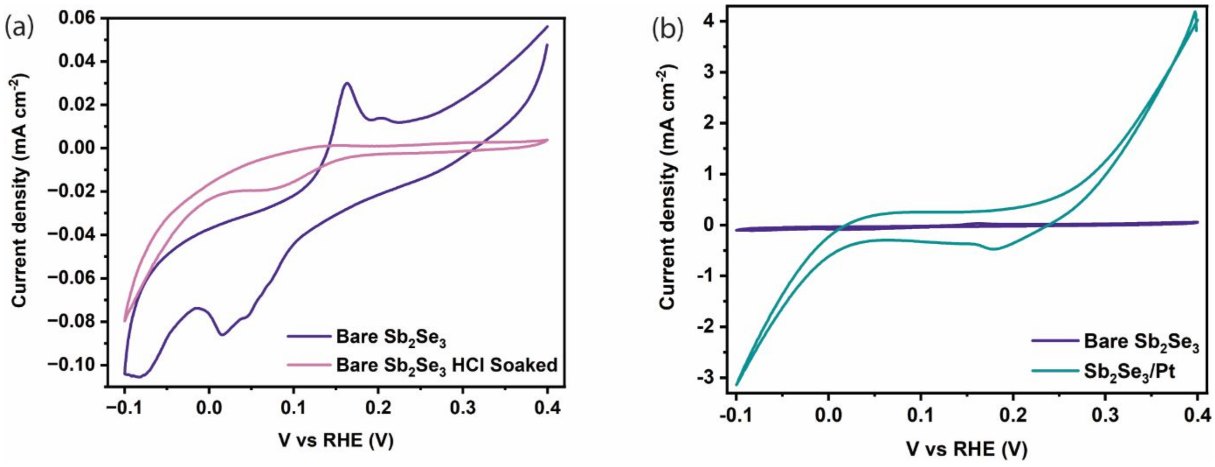

Figure S5 – (a) Cyclic voltammetry measurements of a bare Sb_2Se_3 sample and an Sb_2Se_3 sample soaked in HCl for 12 hours at 1 sun illumination with light chopping. (b) Cyclic voltammetry measurements of a bare Sb_2Se_3 sample and an Sb_2Se_3 sample with 2 nm of Pt as a catalyst at 1 sun illumination with light chopping.

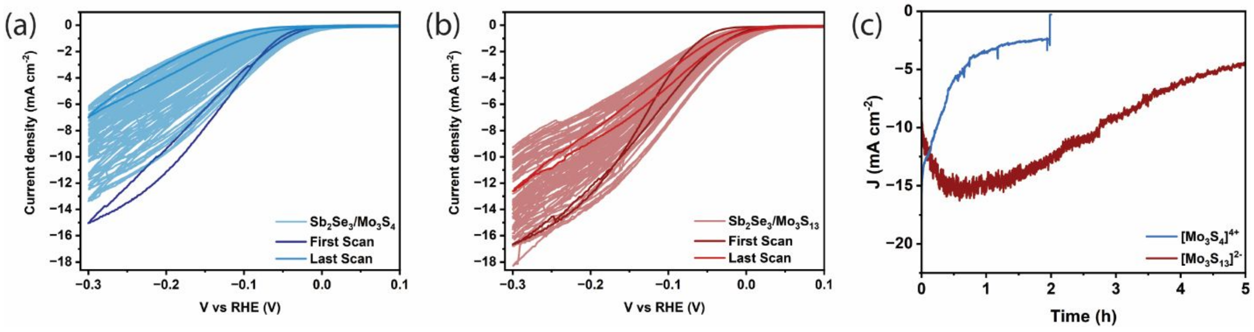

Figure S6 – (a) Cyclic voltammetry measurements of a Sb_2Se_3 + $[Mo_3S_4]^{4+}$ sample for 60 cycles at 1 sun illumination (10 mV s^{-1} equating to 80 minutes). (b) Cyclic voltammetry measurements of a Sb_2Se_3 + $[Mo_3S_{13}]^{2-}$ sample for 60 cycles at 1 sun illumination (10 mV s^{-1} equating to 80 minutes) (c) Chronoamprometric measurements of Sb_2Se_3 + $[Mo_3S_4]^{4+}$ and Sb_2Se_3 + $[Mo_3S_{13}]^{2-}$ at 1 sun illumination.

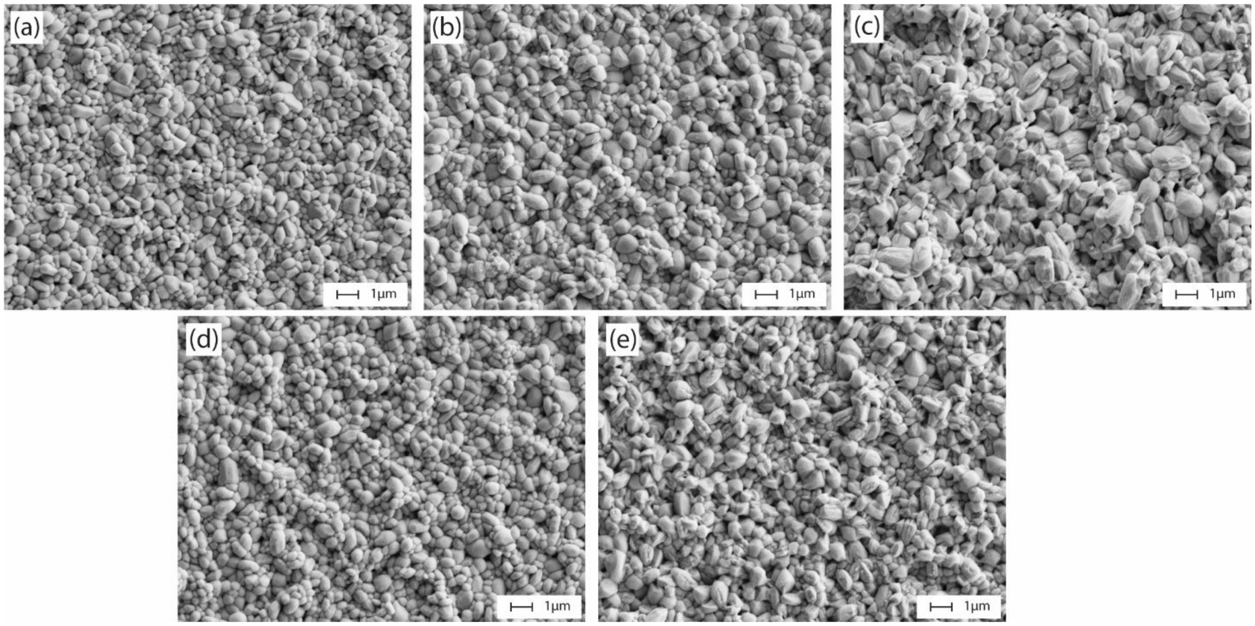

Figure S7 – (a) Top view SEM image of a typical untreated Sb_2Se_3 sample (b) Top view SEM images of a typical Sb_2Se_3 + $[Mo_3S_4]^{4+}$ sample (c) Top view SEM images of a typical Sb_2Se_3 + $[Mo_3S_4]^{4+}$ sample after PEC measurements (d) Top view SEM images of a typical Sb_2Se_3 + $[Mo_3S_{13}]^{2-}$ sample (e) Top view SEM images of a typical Sb_2Se_3 + $[Mo_3S_{13}]^{2-}$ sample after PEC measurements.

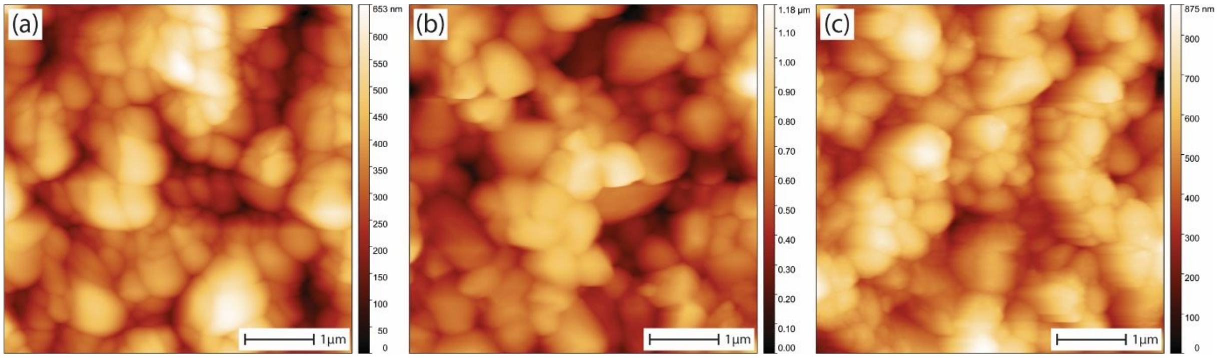

Figure S8 – (a) AFM image of a typical untreated Sb_2Se_3 sample. (b) AFM images of a typical Sb_2Se_3 + $[Mo_3S_4]^{4+}$ sample. (c) AFM images of a typical Sb_2Se_3 + $[Mo_3S_{13}]^{2-}$ sample.

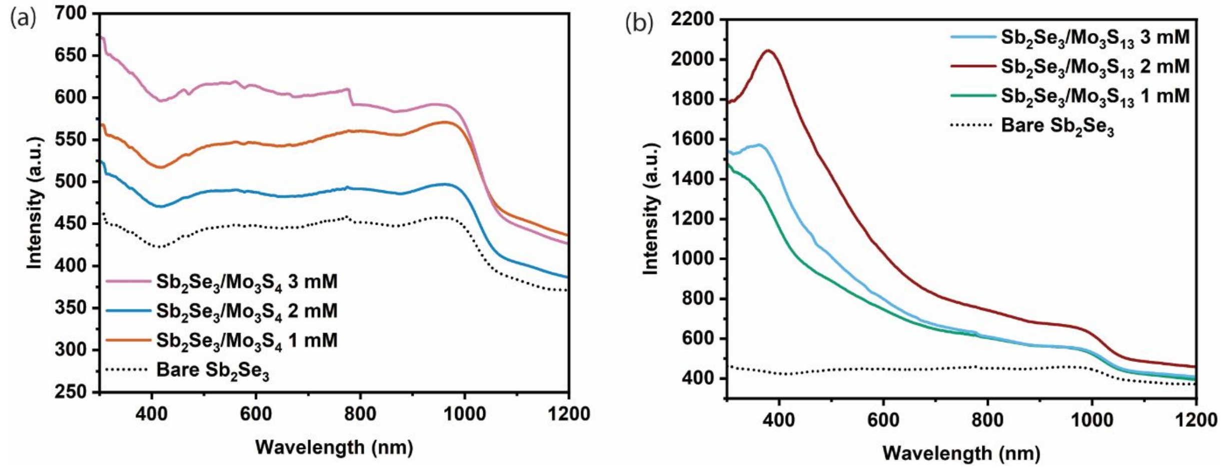

Figure S9 – (a) UV-Vis-NIR DRS of bare Sb_2Se_3 and Sb_2Se_3 + $[Mo_3S_4]^{4+}$ samples at three different concentrations of catalyst deposition solution, 1 mM, 2 mM, 3 mM. (b) Diffuse reflectance spectra of bare Sb_2Se_3 and Sb_2Se_3 + $[Mo_3S_{13}]^{2-}$ samples at three different concentrations of catalyst deposition solution, 1 mM, 2 mM, 3 mM.

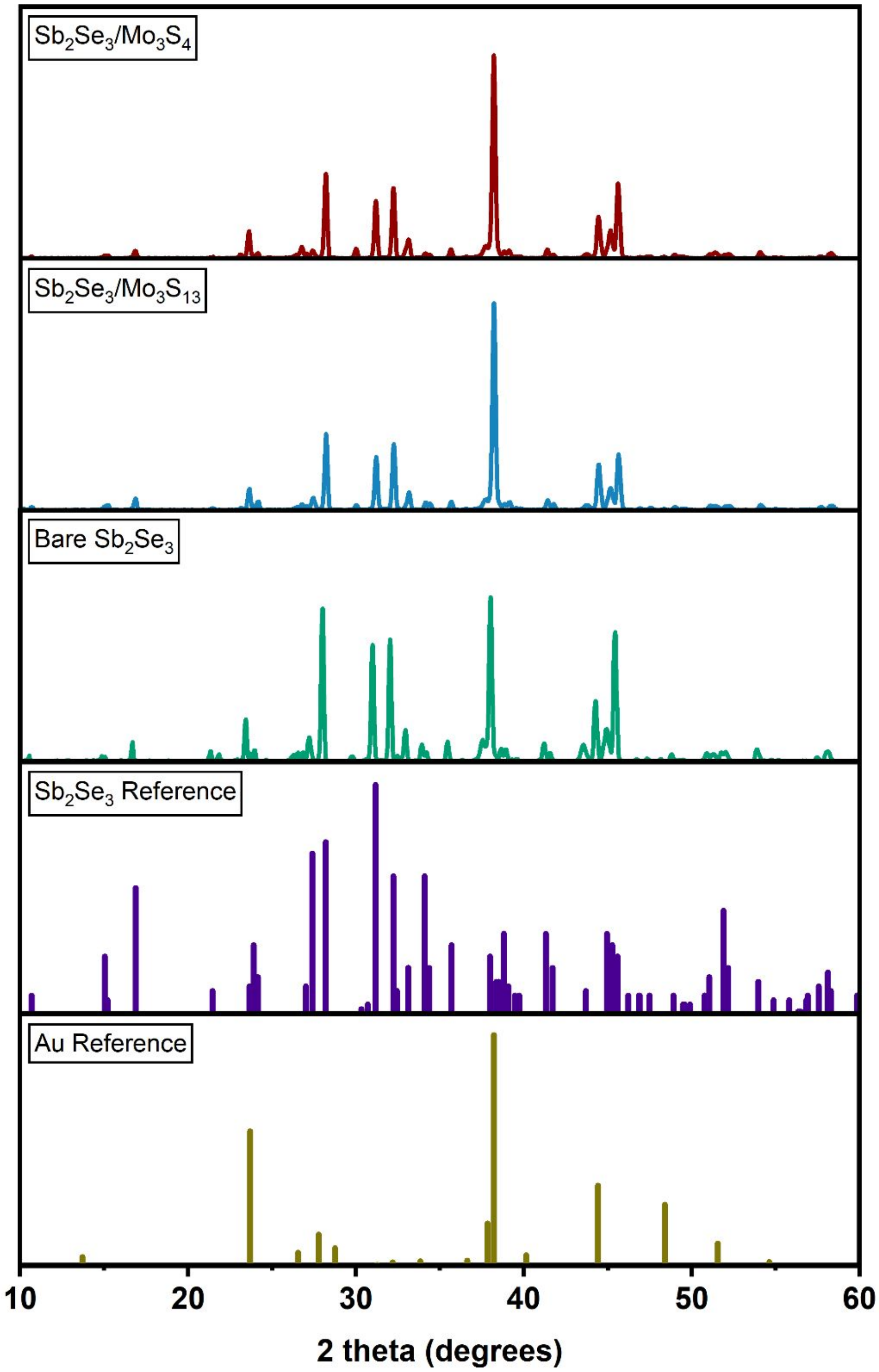

Figure S10 – XRD patterns of bare Sb_2Se_3, Sb_2Se_3 + $[Mo_3S_4]^{4+}$ and Sb_2Se_3 + $[Mo_3S_{13}]^{2-}$ compared to the orthorhombic phase of Sb_2Se_3 and Au reference cards (JCPDS 15-0861 and JCPDS 04-0784 respectively).

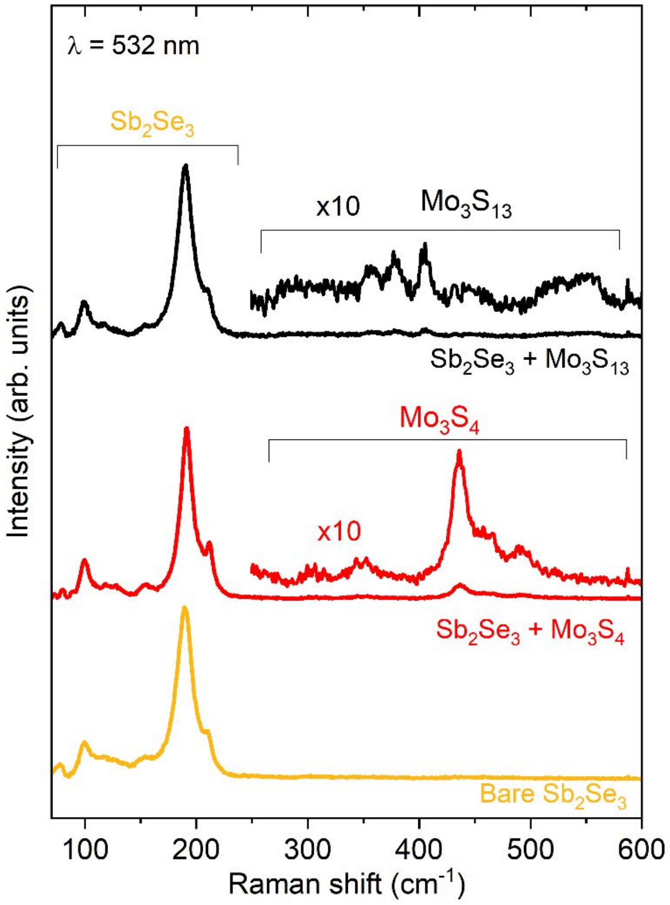

Figure S11 – Raman spectra of a typical bare Sb_2Se_3 sample, Sb_2Se_3 + $[Mo_3S_4]^{4+}$ and Sb_2Se_3 + $[Mo_3S_{13}]^{2-}$ after PEC measurements at the laser excitation wavelengths of 532 nm.

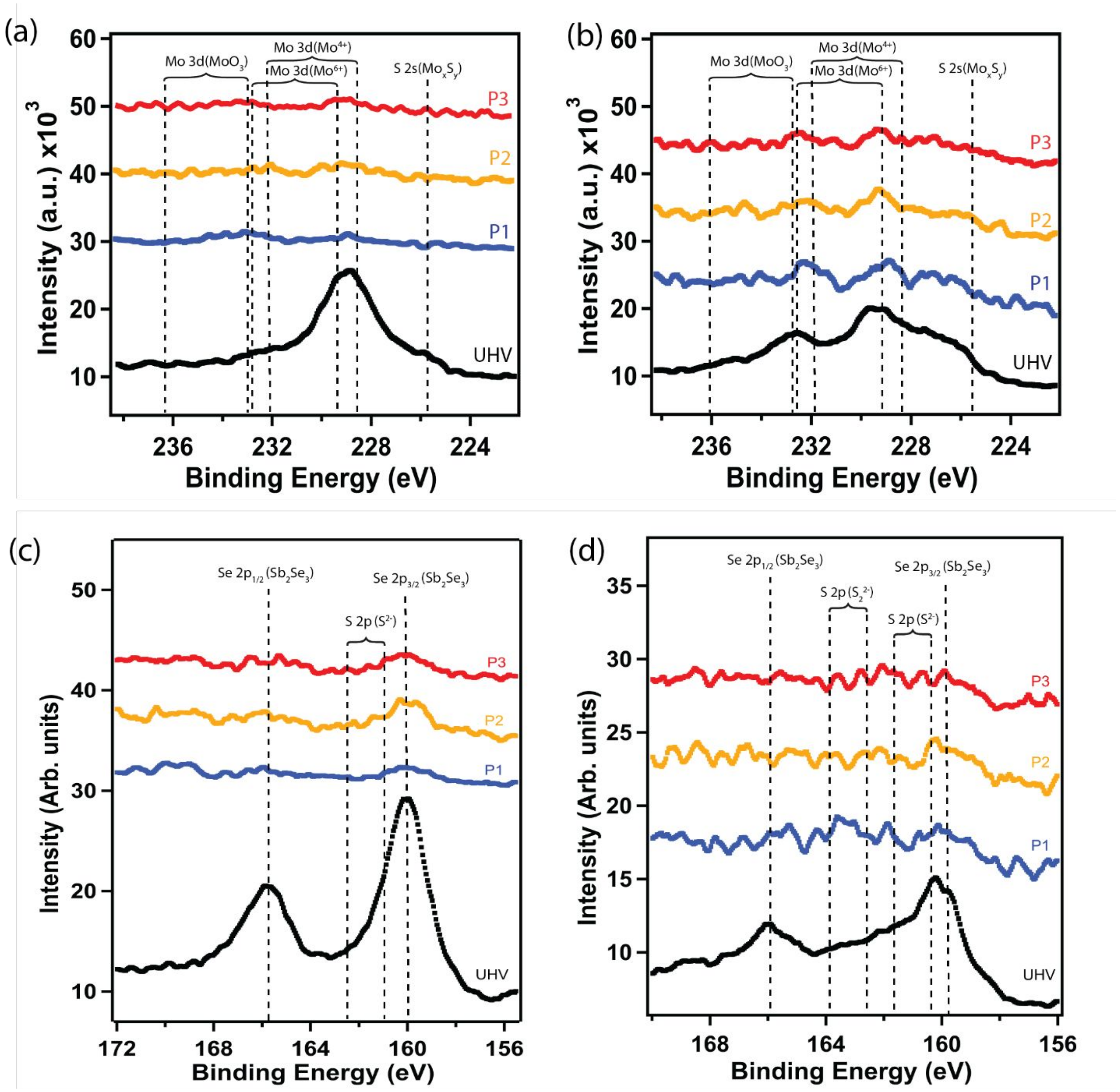

Figure S12 – NAPXPS measurements at a potential before onset (P1 - blue), at a potential around onset (P2 - orange) and at a potential after onset (P3 - red) and at ultra-high vacuum (UHV - black) (a) Mo 3d + S 2s spectrum of Sb_2Se_3 with $[Mo_3S_4]^{4+}$ catalyst (b) Mo 3d + S 2s spectrum of Sb_2Se_3 with $[Mo_3S_{13}]^{2-}$ catalyst (c) Se 3d spectrum of Sb_2Se_3 with $[Mo_3S_4]^{4+}$ catalyst (b) Se 3d spectrum

of Sb_2Se_3 with $[Mo_3S_{13}]^{2-}$ catalyst. An SG 15 function was used to smooth the raw data and the dashed reference lines were extracted from NIST database.[7]

To understand the mechanism of action of the photoabsorber materials and the co-catalyst under realistic conditions such as applied bias, under illumination and in an electrolyte solution, near ambient pressure X-ray photoelectron spectroscopy (NAPXPS) can be utilized. Operating with tender X-rays (~4000 eV), this technique investigates the solid-liquid interface at pressure levels of a few millibars, with the differentially pumped electron analyzer withstanding room-temperature water vapor pressure inside the analysis chamber. Samples with amorphous molybdenum sulfide were highly hydrophilic, stabilizing a thick water film (thickness above 40 nm), which made studies of the solid-liquid interface with NAPXPS impossible. NAPXPS observations deemed the molybdenum sulfide cluster samples suitable, given their stability and minimal hydrophilicity. NAPXPS measurements were designed not only to understand the catalysts' behavior on the surface but also to investigate the charge carrier dynamics of Sb_2Se_3 films through surface photovoltage, which manifests as a change in binding energy. However, no discernible effects or changes were observed at different potentials and between dark and illuminated conditions. Nevertheless, NAPXPS provided valuable insights into the stability of the molybdenum sulfide clusters as catalysts. The NAPXPS endstation at the Swiss Light Source (SLS) at the Paul Scherer Institute (PSI) has a chamber capable of operating up to 30 mbar.[8] In this endstation, it is possible to position a beaker containing an aqueous electrolyte solution just below the inlet cone of the analyzer. After being immersed in the electrolyte, the sample can be set up in a standard three-electrode arrangement to perform in-situ cyclic voltammetry. By employing appropriate reduction-oxidation cycles through electrochemical processes, well-defined surface preparations can be accomplished, resulting in atomically pristine surfaces. The sample can be moved in front of the analyzer cone, maintaining a short distance (600 μm) from the orifice leading to the differentially-pumped lens optics. In instances involving hydrophilic surfaces, sustaining a

continuous water film with a few tens of nanometers thickness is feasible, which allows probing of the liquid-solid interface when using tender X-rays (3-5 keV).[9]

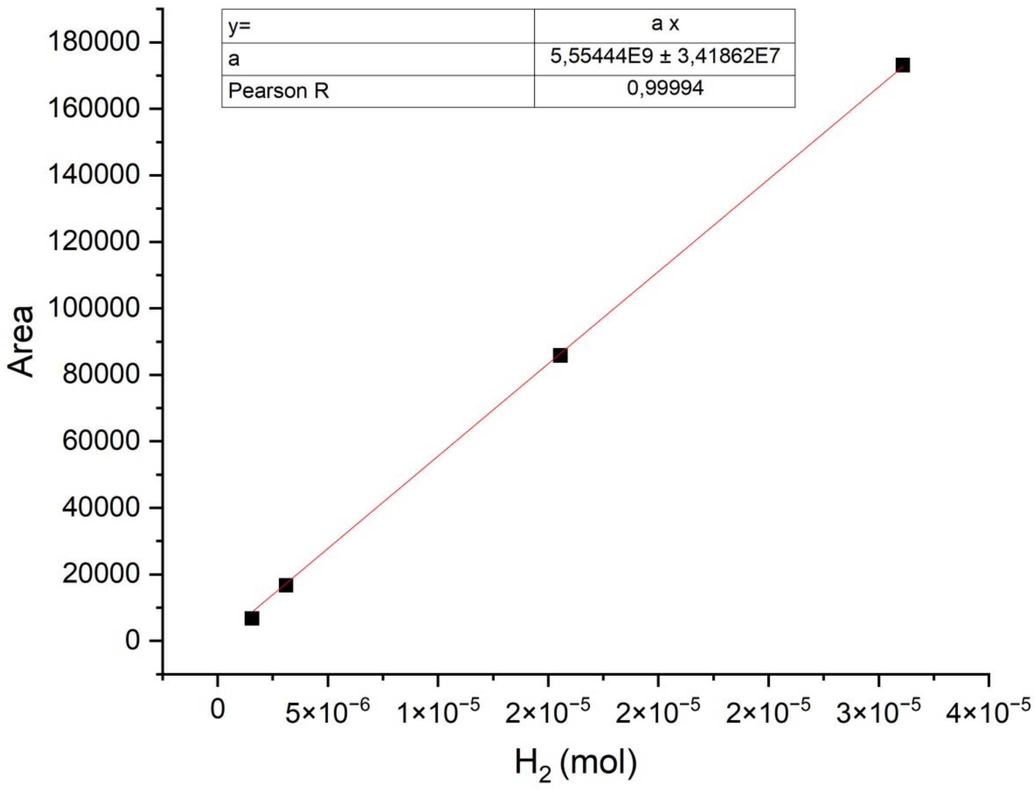

Figure S13 – Calibration curve for the electrochemical hydrogen production and the gas chromatography peak area, assuming a 100% FE for the HER on Pt. The resulting slope of y= a*x is a= $5.55 \cdot 10^9$ (after forcing intercept).

I applied (mA)	t (min)	Q_{el} (C)	N_{H2} (mol)	Area
– 0.5	10	0.300	1.56E-06	6779
– 1	10	0.600	3.11E-06	16744
– 5	10	3.00	1.56E-05	85811
– 10	10	6.00	3.11E-05	173156

Table S5 – Data For Calibration Curve

The calibration was performed by applying galvanostatic currents (–0.5, 1, 5, and 10 mA) to a Pt wire working electrode and a Pt counter electrode in a two-electrode setup, assuming a faradaic efficiency of 100% for HER on Pt. A calibration curve was determined by plotting the peak area measured with gas chromatography versus the produced amount of hydrogen (mol). The produced amount of hydrogen N (mol) was determined via:

$$\mathbf{N = \frac{Q_{el}}{Fn}} \qquad \textbf{(eq. 1)}$$

With Q_{el} = charged flowed during chronopotentiometry (C), n = 2 (number of electrons), F = Faraday constant = 96485.33 (C mol^{-1}). The samples were irradiated with one sun with a white LED, and a potential of –0.2 V versus RHE (chronoamperometry, CA) was applied for specified times. Afterwards, the hydrogen gas was measured. Inserting the measured peak area into the calibration curve gives the hydrogen produced in mol (N).

$$\mathbf{Q = nFN} \qquad \textbf{(eq. 2)}$$

With n = 2 (number of electrons), F = Faraday constant = 96485.33 C mol^{-1}, N produced mol of H_2. Finally, faradaic efficiency was calculated using the equation below:

$$\mathbf{FE = \frac{Q}{Q_{el}} \times 100} \qquad \text{(eq. 3)}$$

Crystal Data

Experimental

Single crystal X-ray diffraction data were collected at 160.0(1) K on a Rigaku OD Supernova/Atlas diffractometer using the copper X-ray radiation (λ = 1.54184 Å) from a dual wavelength X-ray source and an Oxford Instruments Cryojet XL cooler. The selected suitable single crystal was mounted using polybutene oil on a flexible loop fixed on a goniometer head and immediately transferred to the diffractometer. Pre-experiment, data collection, data reduction and analytical absorption correction.[10] were performed with the program suite CrysAlisPro.[11] Using Olex2,[12] the structure was solved with the SHELXt.[13] small molecule structure solution program and refined with the SHELXL2018/3 program package[14] by full-matrix least-squares minimization on F^2.

PLATON[15] was used to check the result of the X-ray analysis. For more details about the data collection and refinement parameters, see the CIF files.

Crystal Data of $[Mo_3S_4(H_2O)_9](OTs)_4$

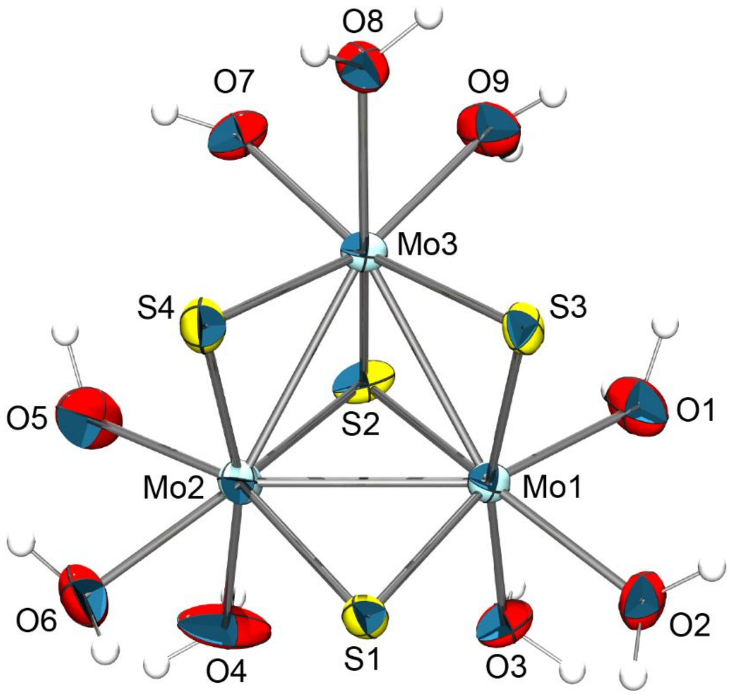

Figure S14 – Displacement ellipsoids of $[Mo_3S_4(H_2O)_9](OTs)_4$. Counter ions and solvent molecules are omitted for clarity, thermal ellipsoids represent 50% probability. Single crystals were obtained after anion exchange with p-toluenesulfonate (OTs) by cooling of a concentrateted solution at −20 °C.[1,2]

Special features

All counterions $C_7H_7O_3S^-$ are partially or completely disordered over two sets of positions. The main species cocrystallized with solvent molecules of water.

Empirical formula	$C_{28}H_{65.72}Mo_3O_{30.86}S_8$
Formula weight	1440.58
Temperature/K	160.0(1)
Crystal system	triclinic
Space group	P-1
a/Å	11.9671(2)
b/Å	15.1770(3)
c/Å	16.5425(3)
α/°	101.825(2)
β/°	96.063(2)
γ/°	109.273(2)
Volume/Å^3	2726.71(9)
Z	2
ρ_{calc}g/cm^3	1.755
μ/mm^{-1}	9.205
F(000)	1469.0
Crystal size/mm^3	0.24 × 0.11 × 0.03

Radiation	Cu Kα (λ = 1.54184)
2Θ range for data collection/°	5.556 to 149.006
Index ranges	$-14 \leq h \leq 14$, $-18 \leq k \leq 18$, $-20 \leq l \leq 20$
Reflections collected	55138
Independent reflections	11106 [R_{int} = 0.0309, R_{sigma} = 0.0204]
Data/restraints/parameters	11106/1907/971
Goodness-of-fit on F^2	1.087
Final R indexes [I>=2σ (I)]	R_1 = 0.0517, wR_2 = 0.1205
Final R indexes [all data]	R_1 = 0.0536, wR_2 = 0.1217
Largest diff. peak/hole / e $Å^{-3}$	1.66/−1.38

Table S6 – Crystal Data and Structure Refinement for $[Mo_3S_4(H_2O)_9](OTs)_4$

Crystal Data for $C_{28}H_{65.72}Mo_3O_{30.86}S_8$ (M =1440.58 g/mol): triclinic, space group P-1 (no. 2), a = 11.9671(2) Å, b = 15.1770(3) Å, c = 16.5425(3) Å, α = 101.825(2)°, β = 96.063(2)°, γ = 109.273(2)°, V = 2726.71(9) $Å^3$, Z = 2, T = 160.0(1) K, μ(Cu Kα) = 9.205 mm^{-1}, Dcalc = 1.755 g/cm^3, 55138 reflections measured (5.556° ≤ 2Θ ≤ 149.006°), 11106 unique (R_{int} = 0.0309, R_{sigma} = 0.0204) which were used in all calculations. The final R_1 was 0.0517 (I > 2σ(I)) and wR_2 was 0.1217 (all data).

Crystal Data of $(NH_4)_2[Mo_3S_{13}]$

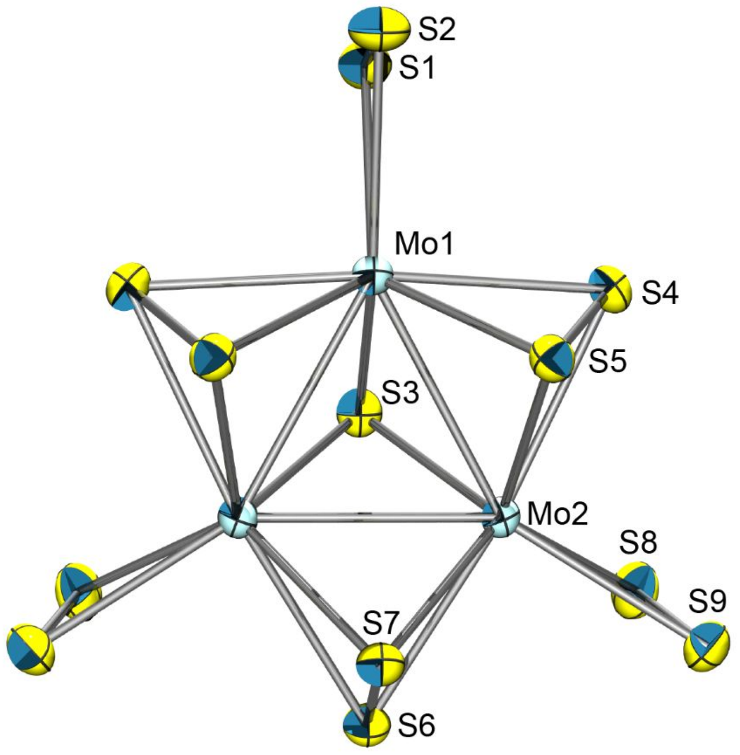

Figure S15 – Displacement ellipsoids of $(NH_4)_2[Mo_3S_{13}]$. Counter ions and solvent molecules are omitted for clarity, thermal ellipsoids represent 50% probability. Single crystals were were picked directly from the obtained crystalline solid after synthesis.[3]

Special features

In the asymmetric unit the $[Mo_3S_{13}]^{2-}$ ion is located on a mirror plane. Only half of the molecule was refined, while the second part being reproduced by a symmetry operation.

Empirical formula	$H_{9.62}Mo_3N_2O_{0.81}S_{13}$
Formula weight	755.28
Temperature/K	160.0(1)
Crystal system	monoclinic
Space group	Cm
a/Å	11.3842(3)
b/Å	16.5251(3)
c/Å	5.68090(10)
α/°	90
β/°	116.354(4)
γ/°	90
Volume/Å^3	957.65(5)
Z	2
ρ_{calc}g/cm^3	2.619
μ/mm^{-1}	29.031
F(000)	728.0
Crystal size/mm^3	0.05 × 0.03 × 0.01

Radiation	Cu Kα (λ = 1.54184)
2Θ range for data collection/°	10.19 to 148.97
Index ranges	−13 ≤ h ≤ 14, −20 ≤ k ≤ 20, −7 ≤ l ≤ 7
Reflections collected	10314
Independent reflections	1946 [R_{int} = 0.0335, R_{sigma} = 0.0223]
Data/restraints/parameters	1946/6/104
Goodness-of-fit on F^2	1.054
Final R indexes [I>=2σ (I)]	R_1 = 0.0212, wR_2 = 0.0539
Final R indexes [all data]	R_1 = 0.0216, wR_2 = 0.0542
Largest diff. peak/hole / e Å^{-3}	1.11/−0.65
Flack parameter	−0.022(11)

Table S7 – Crystal Data and Structure Refinement for $(NH_4)_2[Mo_3S_{13}]$.

Crystal Data for $H_{9.62}Mo_3N_2O_{0.81}S_{13}$ (M =755.28 g/mol): monoclinic, space group Cm (no. 8), a = 11.3842(3) Å, b = 16.5251(3) Å, c = 5.68090(10) Å, β = 116.354(4)°, V = 957.65(5) Å^3, Z = 2, T = 160.0(1) K, μ(Cu Kα) = 29.031 mm^{-1}, Dcalc = 2.619 g/cm^3, 10314 reflections measured (10.19° ≤ 2Θ ≤ 148.97°), 1946 unique (R_{int} = 0.0335, R_{sigma} = 0.0223) which were used in all calculations. The final R_1 was 0.0212 (I > 2σ(I)) and wR_2 was 0.0542 (all data).

Deposition numbers 2320550 (for S14) and 2320551 (for S15) contain the supplementary crystallographic data for this paper. These data are provided free of charge by the joint Cambridge Crystallographic Data Centre and Fachinformationszentrum Karlsruhe Access Structures service.

REFERENCES

(1) Dimitri Coucouvanis. *Inorganic Syntheses*; Coucouvanis, D., Ed.; Inorganic Syntheses; Wiley, 2002; Vol. 33. https://doi.org/10.1002/0471224502.

(2) Shibahara, T.; Yamasaki, M.; Sakane, G.; Minami, K.; Yabuki, T.; Ichimura, A. Syntheses and Electrochemistry of Incomplete Cubane-Type Clusters with M3S4 Cores (M = Molybdenum, Tungsten). X-Ray Structures of [W3S4(H2O)9](CH3C6H4SO3)4.Cntdot.9H2O, Na2[W3S4(Hnta)3].Cntdot.5H2O, and (BpyH)5[W3S4(NCS)9].Cntdot.3H2O. *Inorg Chem* **1992**, *31* (4), 640–647. https://doi.org/10.1021/ic00030a022.

(3) Dave, M.; Rajagopal, A.; Damm-Ruttensperger, M.; Schwarz, B.; Nägele, F.; Daccache, L.; Fantauzzi, D.; Jacob, T.; Streb, C. Understanding Homogeneous Hydrogen Evolution Reactivity and Deactivation Pathways of Molecular Molybdenum Sulfide Catalysts. *Sustain Energy Fuels* **2018**, *2* (5), 1020–1026. https://doi.org/10.1039/c7se00599g.

(4) Adams, P.; Creazzo, F.; Moehl, T.; Crockett, R.; Zeng, P.; Novotny, Z.; Luber, S.; Yang, W.; Tilley, S. D. Solution Phase Treatments of Sb 2 Se 3 Heterojunction Photocathodes for Improved Water Splitting Performance. *J Mater Chem A Mater* **2023**, *11* (15), 8277–8284. https://doi.org/10.1039/D3TA00554B.

(5) Beerbom, M. M.; Lägel, B.; Cascio, A. J.; Doran, B. V.; Schlaf, R. Direct Comparison of Photoemission Spectroscopy and in Situ Kelvin Probe Work Function Measurements on Indium Tin Oxide Films. *J Electron Spectros Relat Phenomena* **2006**, *152* (1–2), 12–17. https://doi.org/10.1016/j.elspec.2006.02.001.

(6) Novotny, Z.; Aegerter, D.; Comini, N.; Tobler, B.; Artiglia, L.; Maier, U.; Moehl, T.; Fabbri, E.; Huthwelker, T.; Schmidt, T. J.; Ammann, M.; van Bokhoven, J. A.; Raabe, J.; Osterwalder, J. Probing the Solid–Liquid Interface with Tender x Rays: A New Ambient-Pressure x-Ray Photoelectron Spectroscopy Endstation at the Swiss Light Source. *Review of Scientific Instruments* **2020**, *91* (2), 023103. https://doi.org/10.1063/1.5128600.

(7) Naumkin, A. V.; Kraut-Vass, A.; Gaarenstroom, S. W.; Powell, C. J.. *NIST X-ray Photoelectron Spectroscopy Database*. https://srdata.nist.gov/xps/ (accessed 2024-05-30).

(8) Novotny, Z.; Aegerter, D.; Comini, N.; Tobler, B.; Artiglia, L.; Maier, U.; Moehl, T.; Fabbri, E.; Huthwelker, T.; Schmidt, T. J.; Ammann, M.; Van Bokhoven, J. A.; Raabe, J.; Osterwalder, J. Probing the Solid-Liquid Interface with Tender x Rays: A New Ambient-Pressure X-Ray Photoelectron Spectroscopy Endstation at the Swiss Light Source. *Review of Scientific Instruments* **2020**, *91* (2), 23103. https://doi.org/10.1063/1.5128600.

(9) Axnanda, S.; Crumlin, E. J.; Mao, B.; Rani, S.; Chang, R.; Karlsson, P. G.; Edwards, M. O. M.; Lundqvist, M.; Moberg, R.; Ross, P.; Hussain, Z.; Liu, Z. Using "Tender" X-Ray Ambient Pressure X-Ray Photoelectron Spectroscopy as A Direct Probe of Solid-Liquid Interface. *Sci Rep* **2015**, *5* (1), 1–12. https://doi.org/10.1038/srep09788.

(10) Clark, R. C.; Reid, J. S. The Analytical Calculation of Absorption in Multifaceted Crystals. *Acta Crystallographica Section A* **1995**, *51* (6), 887–897. https://doi.org/10.1107/S0108767395007367.

(11) *CrysAlisPro*; (version 1.171.43.97a), Rigaku Oxford Diffraction Ltd: Yarnton, Oxfordshire, England, 2023.

(12) Dolomanov, O. V.; Bourhis, L. J.; Gildea, R. J.; Howard, J. A. K.; Puschmann, H. OLEX2: A Complete Structure Solution, Refinement and Analysis Program. *J Appl Crystallogr* **2009**, *42* (2), 339–341. https://doi.org/10.1107/S0021889808042726.

(13) Sheldrick, G. M. SHELXT - Integrated Space-Group and Crystal-Structure Determination. *Acta Crystallogr A* **2015**, *71* (1), 3–8. https://doi.org/10.1107/S2053273314026370.

(14) Sheldrick, G. M. Crystal Structure Refinement with SHELXL. *Acta Crystallogr C Struct Chem* **2015**, *71* (1), 3–8. https://doi.org/10.1107/S2053229614024218.

(15) Spek, A. L. Structure Validation in Chemical Crystallography. *Acta Crystallogr D Biol Crystallogr* **2009**, *65* (2), 148–155. https://doi.org/10.1107/S090744490804362X.
