# Supplementary material for: Direct Anchoring of Molybdenum Sulfide Molecular Catalysts on Antimony Selenide Photocathodes for Solar Hydrogen Production
Source: ACS Energy Lett. 2024 Jul 12;9(8):3828–34. doi: 10.1021/acsenergylett.4c01570 (PMC11320643; doi:10.1021/acsenergylett.4c01570)

checkCIF/PLATON report

Structure factors have been supplied for datablock(s) janb0512

THIS REPORT IS FOR GUIDANCE ONLY. IF USED AS PART OF A REVIEW PROCEDURE FOR PUBLICATION, IT SHOULD NOT REPLACE THE EXPERTISE OF AN EXPERIENCED CRYSTALLOGRAPHIC REFEREE.

No syntax errors found. CIF dictionary Interpreting this report

Datablock: janb0512

Bond precision:	S- S = 0.0025 A		Wavelength=1.54184
Cell:	a=11.3842(3)	b=16.5251(3)	c=5.6809(1)
	alpha=90	beta=116.354(4)	gamma=90
Temperature:	160 K		

	Calculated	Reported
Volume	957.65(5)	957.65(5)
Space group	C m	C 1 m 1
Hall group	C -2y	C -2y
Moiety formula	Mo3 S13, 2(H4 N), 0.802(H2 O)	Mo3 S13, 2(H4 N), 0.802(H2 O)
Sum formula	H9.60 Mo3 N2 O0.80 S13	H9.60 Mo3 N2 O0.80 S13
Mr	755.13	755.28
Dx,g cm-3	2.619	2.619
Z	2	2
Mu (mm-1)	29.031	29.031
F000	728.0	728.0
F000’	736.98	
h,k,lmax		14,20,7
Nref		1946
Tmin,Tmax	0.404,0.748	0.318,0.714
Tmin’	0.204	

Correction method= # Reported T Limits: Tmin=0.318 Tmax=0.714
AbsCorr = ANALYTICAL

Data completeness= Theta(max)= 74.485

R(reflections)= 0.0212(1916) wR2(reflections)= 0.0542(1946)

S = 1.054 Npar= 104

The following ALERTS were generated. Each ALERT has the format
test-name_ALERT_alert-type_alert-level.
Click on the hyperlinks for more details of the test.

Alert level C

```
PLAT077_ALERT_4_C Unitcell Contains Non-integer Number of Atoms ..     Please Check
```

Alert level G

```
PLAT002_ALERT_2_G Number of Distance or Angle Restraints on AtSite          5 Note
PLAT007_ALERT_5_G Number of Unrefined Donor-H Atoms .............          6 Report
              H1A   H1B   H1C   H1D   H1E   H1F
PLAT128_ALERT_4_G Alternate Setting for Input Space Group  Cm             Im Note
PLAT172_ALERT_4_G The CIF-Embedded .res File Contains DFIX Records          2 Report
PLAT302_ALERT_4_G Anion/Solvent/Minor-Residue Disorder (Resd  3  )       100% Note
PLAT304_ALERT_4_G Non-Integer Number of Atoms in ..... (Resd  3  )       1.20 Check
PLAT726_ALERT_2_G H...A   Calc     2.13000, Rep     2.12000 Dev...       0.01 Ang.
              H1D      -O1              1_555   1_555 ........     #    2 Check
PLAT860_ALERT_3_G Number of Least-Squares Restraints ............          6 Note
```

0 **ALERT level A** = Most likely a serious problem - resolve or explain
0 **ALERT level B** = A potentially serious problem, consider carefully
1 **ALERT level C** = Check. Ensure it is not caused by an omission or oversight
8 **ALERT level G** = General information/check it is not something unexpected

0 ALERT type 1 CIF construction/syntax error, inconsistent or missing data
2 ALERT type 2 Indicator that the structure model may be wrong or deficient
1 ALERT type 3 Indicator that the structure quality may be low
5 ALERT type 4 Improvement, methodology, query or suggestion
1 ALERT type 5 Informative message, check

It is advisable to attempt to resolve as many as possible of the alerts in all categories. Often the minor alerts point to easily fixed oversights, errors and omissions in your CIF or refinement strategy, so attention to these fine details can be worthwhile. In order to resolve some of the more serious problems it may be necessary to carry out additional measurements or structure refinements. However, the purpose of your study may justify the reported deviations and the more serious of these should normally be commented upon in the discussion or experimental section of a paper or in the "special_details" fields of the CIF. checkCIF was carefully designed to identify outliers and unusual parameters, but every test has its limitations and alerts that are not important in a particular case may appear. Conversely, the absence of alerts does not guarantee there are no aspects of the results needing attention. It is up to the individual to critically assess their own results and, if necessary, seek expert advice.

Publication of your CIF in IUCr journals

A basic structural check has been run on your CIF. These basic checks will be run on all CIFs submitted for publication in IUCr journals (*Acta Crystallographica*, *Journal of Applied Crystallography*, *Journal of Synchrotron Radiation*); however, if you intend to submit to *Acta Crystallographica Section C* or *E* or *IUCrData*, you should make sure that full publication checks are run on the final version of your CIF prior to submission.

Publication of your CIF in other journals

Please refer to the *Notes for Authors* of the relevant journal for any special instructions relating to CIF submission.

PLATON version of 29/11/2023; check.def file version of 14/09/2023

Datablock janb0512 - ellipsoid plot

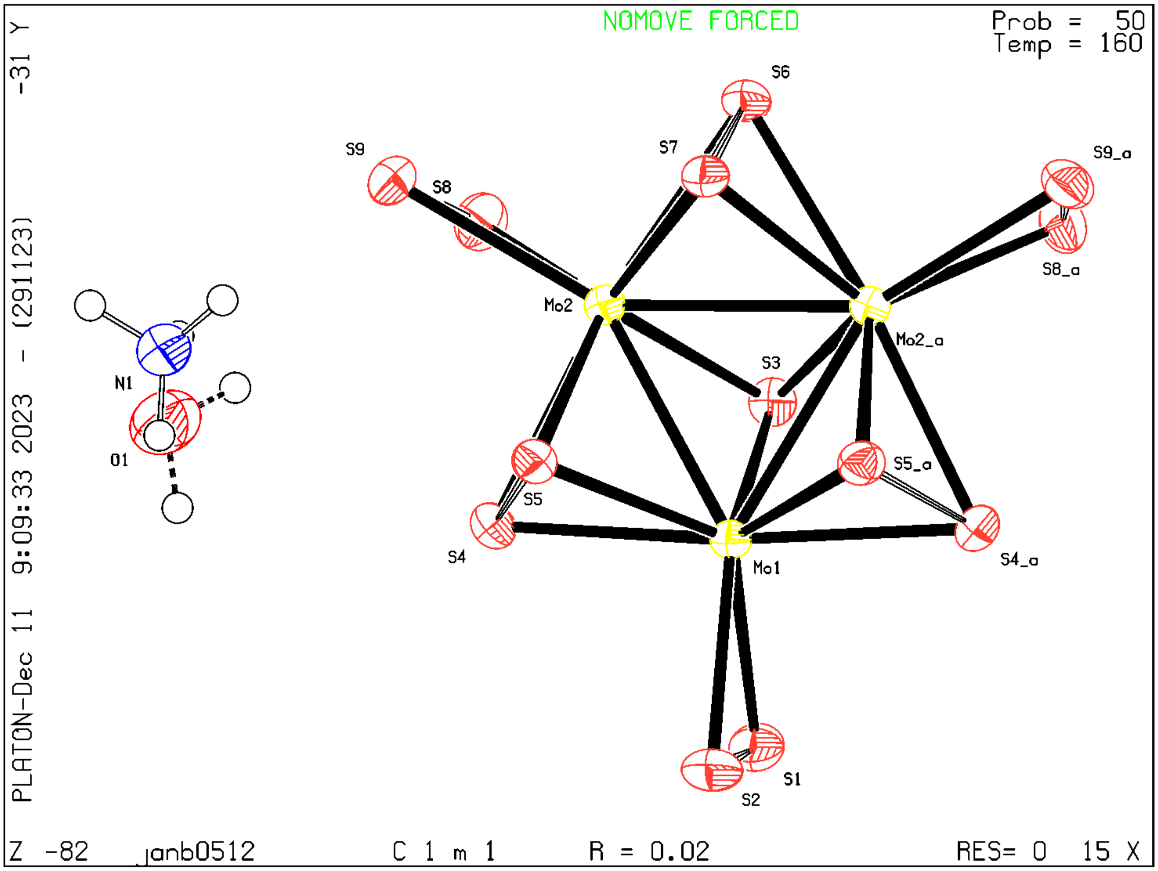

Supplement: Supplementary file 2 — nz4c01570_si_002.zip [file nz4c01570_si_002.zip › Checkcif_Compound 2 1.pdf]
