# Supplementary material for: Direct Anchoring of Molybdenum Sulfide Molecular Catalysts on Antimony Selenide Photocathodes for Solar Hydrogen Production
Source: ACS Energy Lett. 2024 Jul 12;9(8):3828–34. doi: 10.1021/acsenergylett.4c01570 (PMC11320643; doi:10.1021/acsenergylett.4c01570)

checkCIF/PLATON report

Structure factors have been supplied for datablock(s) janb0612

THIS REPORT IS FOR GUIDANCE ONLY. IF USED AS PART OF A REVIEW PROCEDURE FOR PUBLICATION, IT SHOULD NOT REPLACE THE EXPERTISE OF AN EXPERIENCED CRYSTALLOGRAPHIC REFEREE.

No syntax errors found. CIF dictionary Interpreting this report

Datablock: janb0612

```
Bond precision:   C-C = 0.0100 A          Wavelength=1.54184

Cell:           a=11.9671(2)       b=15.1770(3)      c=16.5425(3)
                alpha=101.825(2)   beta=96.063(2)    gamma=109.273(2)
Temperature:    160 K
```

	Calculated	Reported
Volume	2726.71(10)	2726.71(9)
Space group	P -1	P -1
Hall group	-P 1	-P 1
Moiety formula	H18 Mo3 O9 S4, 4(C7 H7 O3 S), 9.838(H2 O)	H18 Mo3 O9 S4, 4(C7 H7 O3 S), 9.838(H2 O)
Sum formula	C28 H65.68 Mo3 O30.84 S8	C28 H65.68 Mo3 O30.84 S8
Mr	1440.19	1440.58
Dx,g cm-3	1.754	1.755
Z	2	2
Mu (mm-1)	9.205	9.205
F000	1468.8	1469.0
F000'	1477.76	
h,k,lmax	14,18,20	14,18,20
Nref	11133	11106
Tmin,Tmax	0.341,0.759	0.217,0.742
Tmin'	0.095	

```
Correction method= # Reported T Limits: Tmin=0.217 Tmax=0.742
AbsCorr = ANALYTICAL

Data completeness= 0.998           Theta(max)= 74.503

R(reflections)= 0.0517( 10634)              wR2(reflections)= 0.1217( 11106)

S = 1.087                  Npar= 971
```

The following ALERTS were generated. Each ALERT has the format
test-name_ALERT_alert-type_alert-level.
Click on the hyperlinks for more details of the test.

Alert level B

PLAT417_ALERT_2_B Short Inter D-H..H-D H1B ..H22B . 2.03 Ang.
x,y,z = 1_555 Check
PLAT417_ALERT_2_B Short Inter D-H..H-D H5A ..H26C . 2.08 Ang.
1-x,-y,-z = 2_655 Check
PLAT417_ALERT_2_B Short Inter D-H..H-D H5A ..H26D . 2.02 Ang.
1-x,-y,-z = 2_655 Check
PLAT420_ALERT_2_B D-H Bond Without Acceptor O36 --H36A . Please Check

Alert level C

PLAT213_ALERT_2_C Atom O4 has ADP max/min Ratio ..... 3.5 prolat
PLAT250_ALERT_2_C Large U3/U1 Ratio for Average U(i,j) Tensor .... 2.6 Note
PLAT250_ALERT_2_C Large U3/U1 Ratio for Average U(i,j) Tensor .... 2.5 Note
PLAT250_ALERT_2_C Large U3/U1 Ratio for Average U(i,j) Tensor .... 2.8 Note
PLAT250_ALERT_2_C Large U3/U1 Ratio for Average U(i,j) Tensor .... 2.4 Note
PLAT334_ALERT_2_C Small <C-C> Benzene Dist. C15A -C20A . 1.37 Ang.
PLAT342_ALERT_3_C Low Bond Precision on C-C Bonds .... 0.01 Ang.
PLAT417_ALERT_2_C Short Inter D-H..H-D H7A ..H36A . 2.10 Ang.
x,y,z = 1_555 Check
PLAT906_ALERT_3_C Large K Value in the Analysis of Variance ...... 3.182 Check
PLAT911_ALERT_3_C Missing FCF Refl Between Thmin & STh/L= 0.600 8 Report
-2 5 0, -2 -3 1, 2 -9 2, 4 -8 2, -2 -7 3, -2 -3 3,
-7 9 3, -7-12 7,
PLAT971_ALERT_2_C Check Calcd Resid. Dens. 0.76Ang From O21A 1.77 eA-3
PLAT975_ALERT_2_C Check Calcd Resid. Dens. 1.08Ang From O36 . 0.83 eA-3
PLAT975_ALERT_2_C Check Calcd Resid. Dens. 0.91Ang From O19A . 0.79 eA-3
PLAT977_ALERT_2_C Check Negative Difference Density on H1A . -0.44 eA-3
PLAT977_ALERT_2_C Check Negative Difference Density on H5A . -0.32 eA-3

Alert level G

PLAT002_ALERT_2_G Number of Distance or Angle Restraints on AtSite 53 Note
PLAT003_ALERT_2_G Number of Uiso or Uij Restrained non-H Atoms ... 65 Report
PLAT007_ALERT_5_G Number of Unrefined Donor-H Atoms .... 48 Report
H1A H1B H2A H2B H3A H3B H4A H4B H5A H5B H6A
H6B H7A H7B H8A H8B H9A H9B H22A H22B H23C H23D
PLAT068_ALERT_1_G Reported F000 Differs from Calcd (or Missing)... Please Check
PLAT083_ALERT_2_G SHELXL Second Parameter in WGHT Unusually Large 21.10 Why ?
PLAT154_ALERT_1_G The s.u.'s on the Cell Angles are Equal ..(Note) 0.002 Degree
PLAT172_ALERT_4_G The CIF-Embedded .res File Contains DFIX Records 8 Report
PLAT173_ALERT_4_G The CIF-Embedded .res File Contains DANG Records 7 Report
PLAT176_ALERT_4_G The CIF-Embedded .res File Contains SADI Records 8 Report
PLAT178_ALERT_4_G The CIF-Embedded .res File Contains SIMU Records 6 Report
PLAT186_ALERT_4_G The CIF-Embedded .res File Contains ISOR Records 1 Report
PLAT188_ALERT_3_G A Non-default SIMU Restraint Value has been used 0.0050 Report
PLAT188_ALERT_3_G A Non-default SIMU Restraint Value has been used 0.0050 Report
PLAT188_ALERT_3_G A Non-default SIMU Restraint Value has been used 0.0050 Report
PLAT188_ALERT_3_G A Non-default SIMU Restraint Value has been used 0.0050 Report
PLAT188_ALERT_3_G A Non-default SIMU Restraint Value has been used 0.0050 Report

```
PLAT188_ALERT_3_G A Non-default SIMU Restraint Value has been used     0.0050 Report
PLAT191_ALERT_3_G A Non-default SADI Restraint Value has been used     0.0050 Report
PLAT191_ALERT_3_G A Non-default SADI Restraint Value has been used     0.0050 Report
PLAT191_ALERT_3_G A Non-default SADI Restraint Value has been used     0.0050 Report
PLAT230_ALERT_2_G Hirshfeld Test Diff for    S7A      --O18A     .        5.5 s.u.
PLAT230_ALERT_2_G Hirshfeld Test Diff for    S8A      --O21A     .       12.7 s.u.
PLAT232_ALERT_2_G Hirshfeld Test Diff (M-X)  Mo2      --S4       .        5.5 s.u.
PLAT232_ALERT_2_G Hirshfeld Test Diff (M-X)  Mo2      --O4       .        6.3 s.u.
PLAT232_ALERT_2_G Hirshfeld Test Diff (M-X)  Mo3      --S4       .        6.8 s.u.
PLAT302_ALERT_4_G Anion/Solvent/Minor-Residue Disorder (Resd  2  )        36% Note
PLAT302_ALERT_4_G Anion/Solvent/Minor-Residue Disorder (Resd  3  )        36% Note
PLAT302_ALERT_4_G Anion/Solvent/Minor-Residue Disorder (Resd  4  )       100% Note
PLAT302_ALERT_4_G Anion/Solvent/Minor-Residue Disorder (Resd  5  )       100% Note
PLAT302_ALERT_4_G Anion/Solvent/Minor-Residue Disorder (Resd  6  )       100% Note
PLAT302_ALERT_4_G Anion/Solvent/Minor-Residue Disorder (Resd  7  )       100% Note
PLAT302_ALERT_4_G Anion/Solvent/Minor-Residue Disorder (Resd  9  )       100% Note
PLAT302_ALERT_4_G Anion/Solvent/Minor-Residue Disorder (Resd 11  )       100% Note
PLAT302_ALERT_4_G Anion/Solvent/Minor-Residue Disorder (Resd 12  )       100% Note
PLAT302_ALERT_4_G Anion/Solvent/Minor-Residue Disorder (Resd 15  )       100% Note
PLAT302_ALERT_4_G Anion/Solvent/Minor-Residue Disorder (Resd 16  )       100% Note
PLAT302_ALERT_4_G Anion/Solvent/Minor-Residue Disorder (Resd 18  )       100% Note
PLAT302_ALERT_4_G Anion/Solvent/Minor-Residue Disorder (Resd 19  )       100% Note
PLAT302_ALERT_4_G Anion/Solvent/Minor-Residue Disorder (Resd 20  )       100% Note
PLAT302_ALERT_4_G Anion/Solvent/Minor-Residue Disorder (Resd 21  )       100% Note
PLAT302_ALERT_4_G Anion/Solvent/Minor-Residue Disorder (Resd 22  )       100% Note
PLAT304_ALERT_4_G Non-Integer Number of Atoms in ..... (Resd  4  )       9.85 Check
PLAT304_ALERT_4_G Non-Integer Number of Atoms in ..... (Resd  5  )       9.79 Check
PLAT304_ALERT_4_G Non-Integer Number of Atoms in ..... (Resd  6  )       8.15 Check
PLAT304_ALERT_4_G Non-Integer Number of Atoms in ..... (Resd  7  )       8.21 Check
PLAT304_ALERT_4_G Non-Integer Number of Atoms in ..... (Resd  9  )       1.53 Check
PLAT304_ALERT_4_G Non-Integer Number of Atoms in ..... (Resd 11  )       1.64 Check
PLAT304_ALERT_4_G Non-Integer Number of Atoms in ..... (Resd 12  )       1.53 Check
PLAT304_ALERT_4_G Non-Integer Number of Atoms in ..... (Resd 15  )       1.63 Check
PLAT304_ALERT_4_G Non-Integer Number of Atoms in ..... (Resd 16  )       1.53 Check
PLAT304_ALERT_4_G Non-Integer Number of Atoms in ..... (Resd 18  )       1.45 Check
PLAT304_ALERT_4_G Non-Integer Number of Atoms in ..... (Resd 19  )       1.17 Check
PLAT304_ALERT_4_G Non-Integer Number of Atoms in ..... (Resd 20  )       1.47 Check
PLAT304_ALERT_4_G Non-Integer Number of Atoms in ..... (Resd 21  )       1.20 Check
PLAT304_ALERT_4_G Non-Integer Number of Atoms in ..... (Resd 22  )       1.37 Check
PLAT333_ALERT_2_G Large Aver C6-Ring C-C Dist C15B     -C20B      .       1.43 Ang.
PLAT415_ALERT_2_G Short Inter D-H..H-X       H28B     ..H36A     .       2.08 Ang.
                                                 1+x,y,1+z  =      1_656 Check
PLAT417_ALERT_2_G Short Inter D-H..H-D       H3A      ..H28G     .       2.00 Ang.
                                                     x,y,z  =      1_555 Check
PLAT417_ALERT_2_G Short Inter D-H..H-D       H3A      ..H28H     .       1.80 Ang.
                                                     x,y,z  =      1_555 Check
PLAT417_ALERT_2_G Short Inter D-H..H-D       H3B      ..H23C     .       1.83 Ang.
                                                     x,y,z  =      1_555 Check
PLAT417_ALERT_2_G Short Inter D-H..H-D       H4B      ..H24D     .       1.83 Ang.
                                                     x,y,z  =      1_555 Check
PLAT417_ALERT_2_G Short Inter D-H..H-D       H5B      ..H33B     .       1.57 Ang.
                                                     x,y,z  =      1_555 Check
PLAT417_ALERT_2_G Short Inter D-H..H-D       H5B      ..H34A     .       2.03 Ang.
                                                     x,y,z  =      1_555 Check
PLAT417_ALERT_2_G Short Inter D-H..H-D       H5B      ..H34B     .       2.11 Ang.
                                                     x,y,z  =      1_555 Check
PLAT417_ALERT_2_G Short Inter D-H..H-D       H7B      ..H35A     .       1.76 Ang.
```

```
                                                 x,y,z  =      1_555 Check
PLAT417_ALERT_2_G Short Inter D-H..H-D       H22B     ..H23C     .       1.55 Ang.
                                                 x,y,z  =      1_555 Check
PLAT417_ALERT_2_G Short Inter D-H..H-D       H23D     ..H26D     .       2.14 Ang.
                                                 x,y,z  =      1_555 Check
PLAT417_ALERT_2_G Short Inter D-H..H-D       H26C     ..H33B     .       1.70 Ang.
                                               1+x,y,z  =      1_655 Check
PLAT417_ALERT_2_G Short Inter D-H..H-D       H26C     ..H34A     .       1.92 Ang.
                                               1+x,y,z  =      1_655 Check
PLAT417_ALERT_2_G Short Inter D-H..H-D       H26C     ..H34B     .       2.09 Ang.
                                               1+x,y,z  =      1_655 Check
PLAT417_ALERT_2_G Short Inter D-H..H-D       H26D     ..H35B     .       1.90 Ang.
                                             1-x,-y,-z  =      2_655 Check
PLAT417_ALERT_2_G Short Inter D-H..H-D       H31B     ..H32A     .       1.57 Ang.
                                            1-x,1-y,-z  =      2_665 Check
PLAT417_ALERT_2_G Short Inter D-H..H-D       H31B     ..H32B     .       1.87 Ang.
                                            1-x,1-y,-z  =      2_665 Check
PLAT720_ALERT_4_G Number of Unusual/Non-Standard Labels ..........         10 Note
              H2AA    H3AA    H2BA    H3BA    H5BA    H6BA    H5AA    H6AA
              H9AA    H9BA
PLAT789_ALERT_4_G Atoms with Negative _atom_site_disorder_group  #          3 Check
PLAT790_ALERT_4_G Centre of Gravity not Within Unit Cell: Resd.  #          2 Note
              C7 H7 O3 S
PLAT790_ALERT_4_G Centre of Gravity not Within Unit Cell: Resd.  #          3 Note
              C7 H7 O3 S
PLAT790_ALERT_4_G Centre of Gravity not Within Unit Cell: Resd.  #          4 Note
              C7 H7 O3 S
PLAT790_ALERT_4_G Centre of Gravity not Within Unit Cell: Resd.  #          5 Note
              C7 H7 O3 S
PLAT790_ALERT_4_G Centre of Gravity not Within Unit Cell: Resd.  #          6 Note
              C7 H7 O3 S
PLAT790_ALERT_4_G Centre of Gravity not Within Unit Cell: Resd.  #          7 Note
              C7 H7 O3 S
PLAT790_ALERT_4_G Centre of Gravity not Within Unit Cell: Resd.  #          8 Note
              H2 O
PLAT790_ALERT_4_G Centre of Gravity not Within Unit Cell: Resd.  #         13 Note
              H2 O
PLAT790_ALERT_4_G Centre of Gravity not Within Unit Cell: Resd.  #         17 Note
              H2 O
PLAT790_ALERT_4_G Centre of Gravity not Within Unit Cell: Resd.  #         22 Note
              H2 O
PLAT811_ALERT_5_G No ADDSYM Analysis: Too Many Excluded Atoms ....          ! Info
PLAT822_ALERT_4_G CIF-embedded .res Contains Negative PART Numbers          1 Check
PLAT860_ALERT_3_G Number of Least-Squares Restraints .............       1907 Note
PLAT912_ALERT_4_G Missing # of FCF Reflections Above STh/L=  0.600         19 Note
PLAT933_ALERT_2_G Number of HKL-OMIT Records in Embedded .res File          6 Note
               -2 -7  3,  -2 -3  1,  -2 -3  3,  -2  5  0,   2 -9  2,   4 -8  2,
PLAT978_ALERT_2_G Number C-C Bonds with Positive Residual Density.          9 Info
```

---

  0 **ALERT level A** = Most likely a serious problem - resolve or explain
  4 **ALERT level B** = A potentially serious problem, consider carefully
 15 **ALERT level C** = Check. Ensure it is not caused by an omission or oversight
 91 **ALERT level G** = General information/check it is not something unexpected

  2 ALERT type 1 CIF construction/syntax error, inconsistent or missing data

```
44 ALERT type 2 Indicator that the structure model may be wrong or deficient
13 ALERT type 3 Indicator that the structure quality may be low
49 ALERT type 4 Improvement, methodology, query or suggestion
 2 ALERT type 5 Informative message, check
```

It is advisable to attempt to resolve as many as possible of the alerts in all categories. Often the minor alerts point to easily fixed oversights, errors and omissions in your CIF or refinement strategy, so attention to these fine details can be worthwhile. In order to resolve some of the more serious problems it may be necessary to carry out additional measurements or structure refinements. However, the purpose of your study may justify the reported deviations and the more serious of these should normally be commented upon in the discussion or experimental section of a paper or in the "special_details" fields of the CIF. checkCIF was carefully designed to identify outliers and unusual parameters, but every test has its limitations and alerts that are not important in a particular case may appear. Conversely, the absence of alerts does not guarantee there are no aspects of the results needing attention. It is up to the individual to critically assess their own results and, if necessary, seek expert advice.

Publication of your CIF in IUCr journals

A basic structural check has been run on your CIF. These basic checks will be run on all CIFs submitted for publication in IUCr journals (*Acta Crystallographica*, *Journal of Applied Crystallography*, *Journal of Synchrotron Radiation*); however, if you intend to submit to *Acta Crystallographica Section C* or *E* or *IUCrData*, you should make sure that full publication checks are run on the final version of your CIF prior to submission.

Publication of your CIF in other journals

Please refer to the *Notes for Authors* of the relevant journal for any special instructions relating to CIF submission.

PLATON version of 29/11/2023; check.def file version of 14/09/2023

Datablock janb0612 - ellipsoid plot

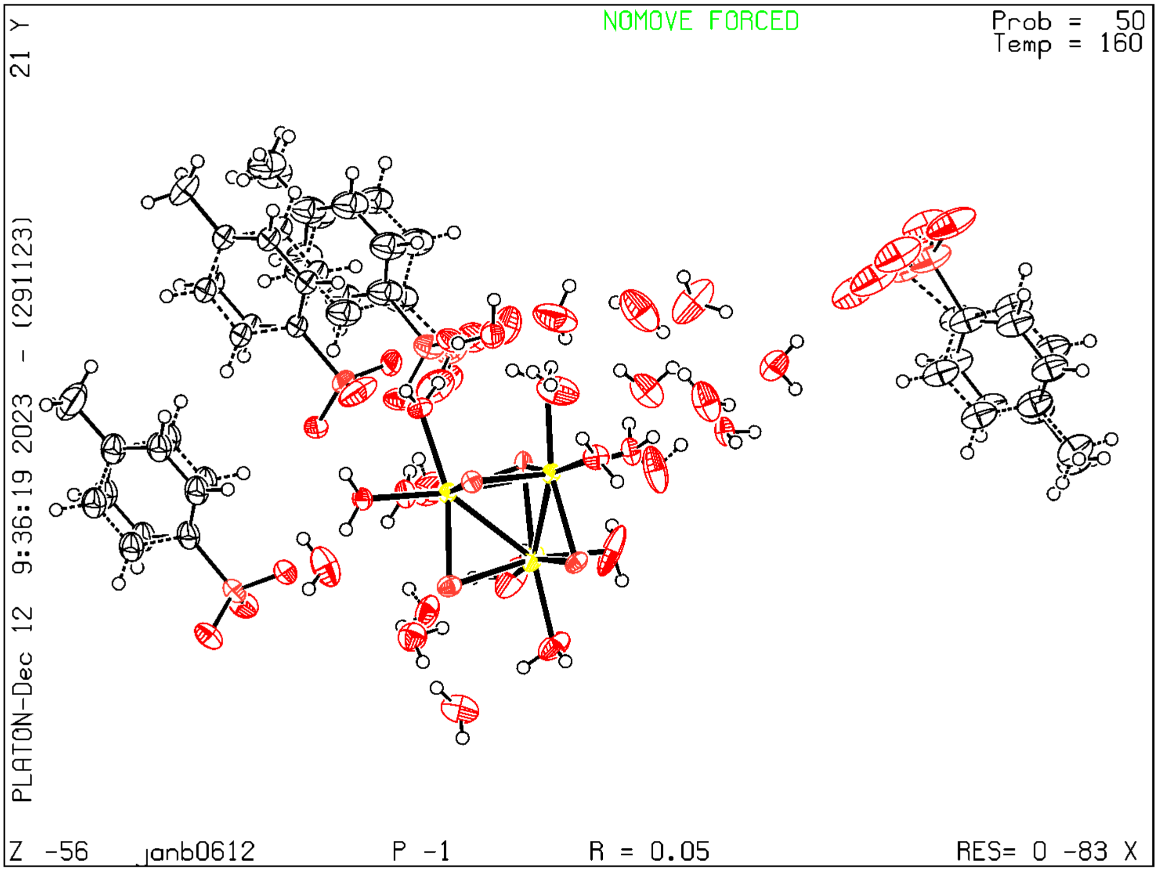

Supplement: Supplementary file 2 — nz4c01570_si_002.zip [file nz4c01570_si_002.zip › Checkcif_Compound 1 1.pdf]
